# Transcriptome assembly in *Suaeda aralocaspica* to reveal the distinct temporal gene/miRNA alterations between the dimorphic seeds during germination

**DOI:** 10.1186/s12864-017-4209-1

**Published:** 2017-10-19

**Authors:** Lei Wang, Hong-Ling Wang, Lan Yin, Chang-Yan Tian

**Affiliations:** 1 State Key Laboratory of Desert and Oasis Ecology, Xinjiang Institute of Ecology and Geography, Chinese Academy of Sciences, Urumqi, 830011 China; 2 ABLife, Inc., Optics Valley International Biomedical Park, Building 18, East Lake High-Tech Development Zone, 858 Gaoxin Boulevard, Wuhan, 430075 China

**Keywords:** *Suaeda aralocaspica*, Euhalophyte, De novo assembly, Transcriptome, Dimorphic seed, Non-dormant, Non-deep dormant, Germination

## Abstract

**Background:**

Dimorphic seeds from *Suaeda aralocaspica* exhibit different germination behaviors that are thought to be a bet-hedging strategy advantageous in harsh and unpredictable environments. To understand the molecular mechanisms of *Suaeda aralocaspica* dimorphic seed germination, we applied RNA sequencing and small RNA sequencing for samples collected at three germination stages.

**Results:**

A total of 79,414 transcripts were assembled using Trinity, of which 57.67% were functionally annotated. KEGG enrichment unveiled that photosynthesis and flavonol biosynthesis pathways were activated earlier in brown seed compared with black seed. Gene expression analysis revealed that nine candidate unigenes in gibberellic acid and abscisic acid signal transduction and 23 unigenes in circadian rhythm-plant pathway showed distinct expression profiles to promote dimorphic seed germination. 194 conserved miRNAs comprising 40 families and 21 novel miRNAs belonging to 20 families in *Suaeda aralocaspica* were identified using miRDeep-P and Mfold. The expression of miRNAs in black seed was suppressed at imbibition stage. Among the identified miRNAs, 59 conserved and 13 novel miRNAs differentially expressed during seed germination. Of which, 43 conserved and nine novel miRNAs showed distinct expression patterns between black and brown seed. Using TAPIR, 208 unigenes were predicted as putative targets of 35 conserved miRNA families and 17 novel miRNA families. Among functionally annotated targets, genes participated in transcription regulation constituted the dominant category, followed by genes involved in signaling and stress response. Seven of the predicted targets were validated using 5′ rapid amplification of cDNA ends or real-time quantitative reverse transcription-PCR.

**Conclusions:**

Our results indicate that specific genes and miRNAs are regulated differently between black and brown seed during germination, which may contribute to the different germination behaviors of *Suaeda aralocaspica* dimorphic seeds in unpredictable variable environments. Our results lay a solid foundation for further studying the roles of candidate genes and miRNAs in *Suaeda aralocaspica* dimorphic seed germination.

**Electronic supplementary material:**

The online version of this article (10.1186/s12864-017-4209-1) contains supplementary material, which is available to authorized users.

## Background

*Suaeda aralocaspica* is a monoecious annual central Asian halophyte, which is commonly found in saline-alkaline sandy soils of Gobi desert. In China, *S. aralocaspica* is restricted to the inland cold desert of the Junggar Basin, Xinjiang. Desert annuals are known to have well-developed seed dispersal and germination mechanisms to survive the harsh environment [1, 2]. *S. aralocaspica* produces two distinct types of seeds that differ in morphology, dormancy and germination characteristics [3]. Oblate brown seeds covered with a soft seed coat are highly permeable to water and exhibit non-dormant behavior. Brown seeds can germinate rapidly to high percentages over a wide range of temperature regimes in both white light and darkness. In contrast, elliptical black seeds are covered with a rigid seed coat. Although this type of seeds also take up water, they germinate slowly to low percentages in various viability testing conditions [3]. An imbibed viable seed being not able to germinate under favorable conditions is dormancy [4–7]. We, thereafter, call the black seeds the non-deep dormant seeds [4]. *S. aralocaspica* developing a unique combination of dispersal and germination strategies via producing dimorphic seeds is thought to be a bet-hedging strategy advantageous in harsh and unpredictable environments [3, 8–10].

Germination is a critical phase in the plant life cycle. Generally this process starts with the uptake of water by the dry mature seed. Upon imbibition of water, the dry mature seed swell and enzymes and food supplies become hydrated. Hydration re-initiates the metabolic activities in seed to produce energy for growth process. Genome-wide expression studies in *Arabidopsis thaliana* have been previously applied to gain insight into temporal and spatial changes during Arabidopsis germination and provide important new information about mechanisms controlling germination [11–15]. Nevertheless, a detailed knowledge of the temporal alterations in gene/miRNA in halophyte seed is far missing. In order to understand the control of the timing of germination as well as the underlying molecular processes contributed by the bet-hedging germination, we analyzed *S. aralocaspica* transcriptome by sampling three points along the germination time course. Our high throughput data set will shed light on the temporal alterations occur during dimorphic seed germination of *S. aralocaspica* and provide a comprehensive list of candidate genes and miRNAs showing potential regulatory mechanisms during this process.

## Results

### Morphology of *S. aralocaspica* seed germination

In *S. aralocaspica*, seed germination is a process of spiral embryos uncoiling. Black (Bl) dry seeds (DS) had thinly leathery testae, brown (Br) DSs were covered with membranous seed coat (Additional file 1: Figure S1A). At imbibed seed (IS) stage, brown seed absorbed water initially via seed coat and black seed via testae. Along with the imbibition of water, DSs swelled, the seed coat of BrDS stretched while the testa of BlDS cracked (Additional file 1: Figure S1B). At seedling (S) stage, both embryos were uncoiled, the one of brown seed untwisted faster than that of black seed (Additional file 1: Figure S1C).

### RNA sequence (RNA-seq) and filter

A total of 225,861,504 raw reads of 100 bp were generated from BlDS, BlIS, BlS, BrDS, BrIS, and BrS cDNA libraries. After removal of low-quality reads, a total of 201,609,259 high-quality reads were identified, which contained 17,011,844,081 nucleotides. The average length of the reads is 84 base pairs and the percentage of Q20 bases (base quality more than 20 and an error rate of less than 0.01) is 97.43% (Additional file 2: Table S1 and S2).

### De novo transcriptome assembly

Previous studies have documented that Trinity was a special short-read assembly method for the efficient de novo reconstruction of the transcriptome, and 25-mer was the optimal parameter [16–18]. In this study, the clean reads were assembled de novo using Trinity with a 25-mer parameter and generated 106,171 transcripts. Then, Bowtie 2 [19] was introduced to remove the false positive transcripts, the total transcripts were reduced by 9831. Mapping coverage for this assembly was 83.15%. To reduce the assembly redundancy, we ran the clustering methods using CD-HIT [20] on the assembly. At last, we obtained 79,414 non-redundant transcripts with a total of 51,415,356 nucleotides. The average length and N50 length of these transcripts were 647 bp and 963 bp, respectively (Additional file 2: Table S2). The sequencing coverage depth range from 0.9907- to 392,592.2545-fold and the median fold is 16.9680. Of 79,414 high-quality transcripts, 32,381 (40.77%) are longer than 500 bp, 15,192 (19.13%) are longer than 1000 bp, and 3151 (3.97%) are longer than 2000 bp.

### Functional annotation and coding region sequences (CDS) prediction

The sequences of *S. aralocaspica* transcripts were searched against the non-redundant (Nr), clusters of orthologous groups (COG), Swiss-Prot and Kyoto Encyclopedia of Genes and Genome (KEGG) protein databases, a total of 44,327 transcripts (55.82%) were annotated in these four databases (Additional file 2: Table S3). *B. vulgaris* is the only species that has been sequenced in the family of *Chenopodiaceae*. *Populus euphratica* is a halophyte poplar species growing in saline semi-arid areas. There were 44,041 transcripts of *S. aralocaspica* realigned to the *B. vulgaris* genome; and 36,050 transcripts realigned to *P. euphratica* genome (Additional file 2: Table S3). In total, 45,796 transcripts (57.67%) were aligned to homologous sequences in public databases, *B. vulgaris* genome, *P. euphratica* genome. A large proportion had no significant sequence alignment or hits in any of the databases, which suggested that they might contain novel sequences or a high number of special genes in to *S. aralocaspica*.

Using BLAST2GO [21] program, we obtained gene ontology (GO) functional annotations of the *S. aralocaspica* transcripts with Nr annotations. A total of 13,563 transcripts were identified with significant enrichment (*p*-value <0.05) (Additional file 3: Figure S2). In the biological process category, “metabolic process”, “oxidation reduction”, “regulation of transcription, DNA-dependent” were the three dominant subcategories. In the other two main categories, the most prominent subcategories were “ATP binding” and “integral to membrane”, respectively.

Based on the four public protein databases, we obtained a total of 36,531 CDSs (26,017 CDSs predicted by the BLAST search and 10,514 by ESTScan). As shown in Additional file 4: Figure S3, 39,496 transcripts less than 400 bp were not well annotated. The transcripts not predicted with a CDS were likely either too short to meet the criterion of CDS prediction or were non-coding RNAs.

### Identification of differentially expressed genes (DEGs) during seed germination

To acquire counts data for differential expression analysis, clean reads generated from different stages (DS, IS, S) were mapped to the newly generated reference transcriptome using Bowtie 2 [19]. The reads per kilobase of transcript per million mapped reads (RPKM) value and number of transcripts for each gene were summarized in Additional file 5: Table S4.

**Fig. 1:**
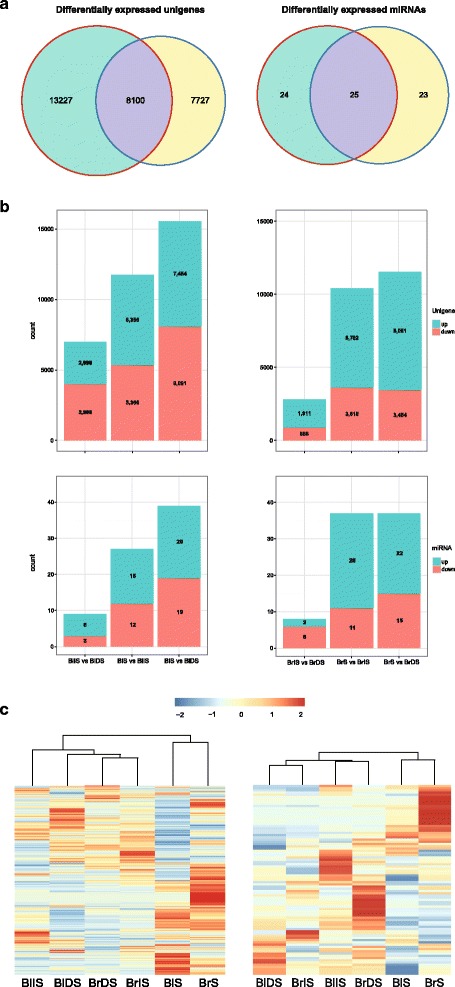
Comparative mRNA and miRNA transcriptome of *Suaeda aralocaspica* between black and brown seed during germination. **a** Venn diagram of the number of differentially expressed unigenes (left) and miRNAs (right) in black (green) and brown (yellow) seed germination. **b** The number of differentially expressed unigenes (upper) and miRNAs (lower) in black (left) and brown (right) seeds. Aquamarine color bars refer to up-regulated unigenes/miRNAs, salmon color bars refer to down-regulated unigenes/miRNAs. **c** Expression of the differentially expressed unigenes (left) and miRNAs (right) identified in *Suaeda aralocaspica* seed germination. BlDS represents black dry seed, BlIS represents black imbibed seed, BlS represents seedlings germinated from black seed, BrDS represents brown dry seed, BrIS represents brown imbibed seed, BrS represents seedlings germinated from brown seed

Calculated read number was directly used for comparing the differences in gene counts between any two germination stages using EdgeR [22]. As shown in Fig. 1A, more DEGs were identified in black seed (21,327 DEGs) than brown seed (15,827 DEGs) during *S. aralocaspica* germination (Additional file 6: Table S5). Among them, 8100 DEGs were developmentally regulated in both seed, these DEGs were possibly involved in the common biological processes for both seed during *S. aralocaspica* germination. The number of up-regulated and down-regulated genes in IS vs. DS, S vs. IS, and S vs. DS was displayed in Fig. 1B. Notably, in the comparison between DS and IS stages, the number of DEGs in brown seed was smaller than that of black seed, however, in the comparison between IS and S stages, the number of DEGs in brown seed was dramatically increased and similar to the number of DEGs in black seed. The heatmap of DEGs illustrated that the expression profiles of BrDS and BrIS were more similar when comparing to the expression profiles of BlDS and BlIS, whereas BlS and BrS formed a tight cluster with a distinct pattern of gene expression (Fig. 1C). These findings indicated that a larger number of DEGs participated in black seed germination comparing to brown seed, and the difference in the number of DEGs between black and brown seed was most likely attributed to the dormancy breaking required for black seed germination.

**Fig. 2:**
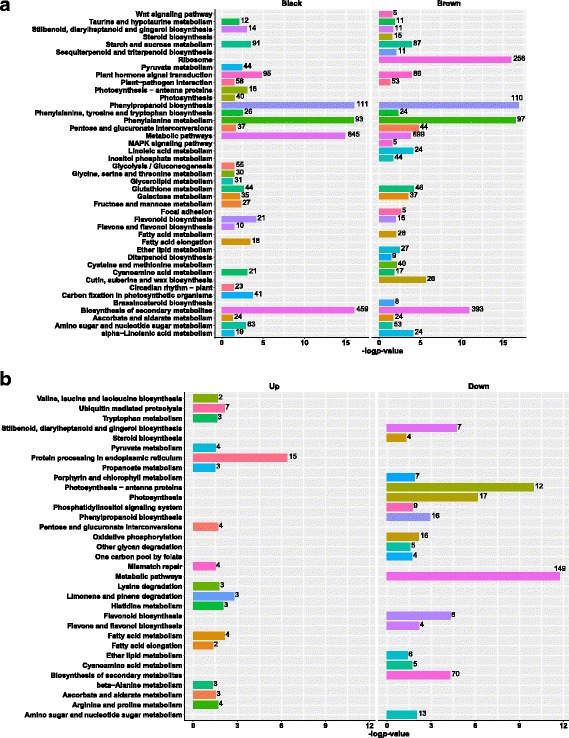
The significantly overrepresented KEGG pathways identified by enrichment analysis for differentially expressed unigenes. **a** Differentially expressed unigenes during *Suaeda aralocaspica* dimorphic seed germination. **b** Differentially expressed unigenes in the comparison between black and brown dry seed. X-axis represents the base 10 logarithm of the enrichment *p*-value, y-axis represents the term of enriched KEGG pathways. The number of differentially expressed unigenes in each pathway is indicated at the end of each bar. Up means up-regulated unigenes in black dry seed, Down means down-regulated unigenes in black dry seed

KEGG enrichment was performed to identify pathways that were related to *S. aralocaspica* seed germination. Enrichment analysis identified 29 pathways in black seed and 32 pathways in brown seed that were significantly overrepresented (*p*-value <0.05, Fig. 2A, Additional file 7: Table S6). There were 18 enriched pathways were overrepresented in both black and brown seed, indicating these 18 KEGG pathways were possibly common biological processes for *S. aralocaspica* seed germination.

### Identification of DEGs between black and brown dry seed

To gain an insight into the different biological processes occurred inside the black and brown dry seed, the gene expression levels between BlDS and BrDS were analyzed (Additional file 8: Table S7). In comparison with BrDS, there were 2420 genes up-regulated and 1846 genes down-regulated in BlDS (*p*-value <0.01, |log_2_ fold change (FC)| ≥1). KEGG enrichment analysis identified 16 significantly overrepresented pathways for up-regulated genes and 17 pathways for down-regulated genes (*p*-value <0.05, Fig. 2B, Additional file 9: Table S8).

### The genes involved in gibberellic acid (GA) and abscisic acid (ABA) signal transduction

**Fig. 3:**
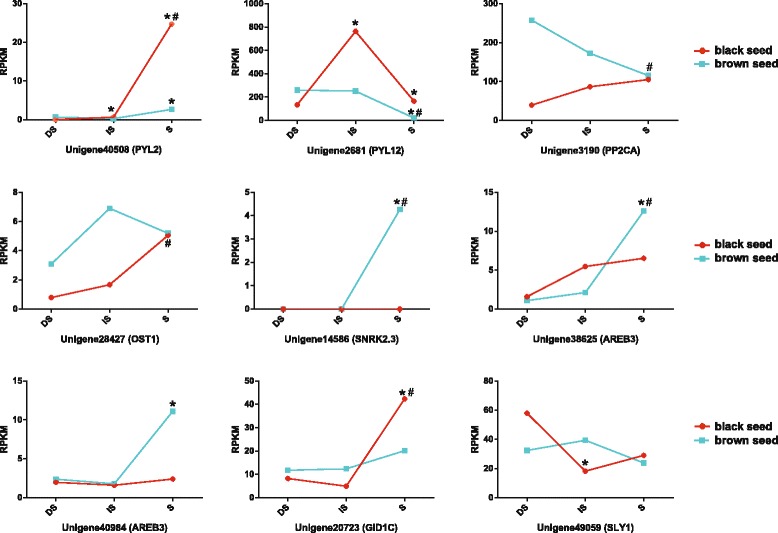
Distinct expression patterns of abscisic acid and gibberellic acid-related genes between black and brown seed. Red lines represent the transcript levels in black seed, green lines represent the transcript levels in brown seed. The transcript levels were determined by reads per kilobase of transcript per million mapped reads (RPKM). DS represents dry seed, IS represents imbibed seed, S represents seedlings of *Suaeda aralocaspica*. * means significant differences (*p* < 0.05) in IS vs. DS or S vs. IS, and **#** means significant differences (*p* < 0.05) in S vs. DS. The abbreviations of abscisic acid/gibberellic acid gene names were listed with parentheses

The balance of GA: ABA levels and sensitivity have shown to be important factors in the regulation of seed germination [23–25]. In this study, 41 GA or ABA signal genes (57 transcripts) were identified. Among them, 26 unigenes showed differential expression patterns at least in one type of seed germination (Additional file 10: Table S9). Most GA or ABA signal DEGs exhibited similar temporal changes in the expression levels in both seed, indicating these signal DEGs may exert the same functions in black and brown seed germination. However, transcript levels of nine GA or ABA signal genes showed distinct expression patterns between black and brown seed during germination (Fig. 3). The transcript level of PYL2 (Unigene40508) was drastically increased at S stage in black seed, whereas slightly enhanced in brown seed. The level of PYL12 (Unigene2681) was raised rapidly then reduced markedly in black seed, while continuously dropped to a low level in brown seed. The expressions of PP2CA (Unigene3190) and SLY1 (Unigene49059) in black and brown seed were changed in the opposite directions. The transcript level of OST1 (Unigene28427) was continuously enhanced in black seed, whereas was increased at first, then decreased in brown seed. The expression of SNRK2.3 (Unigene14586) was only detected at S stage in brown seed. AREB3 (Unigene38625 and Unigene40984) showed no significant change in expression levels in black seed, but exhibited a dramatic rising at S stage in brown seed. The level of GID1C (Unigene20723) was slightly decreased then markedly increased in black seed, but gradually enhanced in brown seed.

### DEGs involved in plant circadian clock

**Fig. 4:**
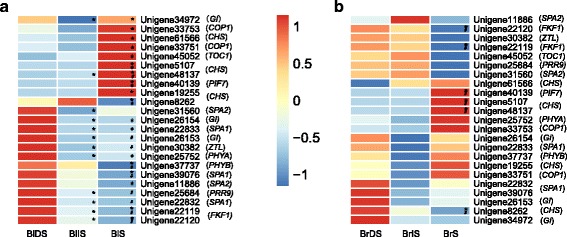
Heat maps of 23 clock genes in black (**a**) and brown (**b**) seed during germination. In the heatmaps, RPKM (reads per kilobase of transcript per million mapped reads) value of each gene was replaced by log_2_RPKM. Colors ranged from blue to red, corresponding to low to high expressions. BlDS represents black dry seed, BlIS represents black imbibed seed, BlS represents seedlings germinated from black seed, BrDS represents brown dry seed, BrIS represents brown imbibed seed, BrS represents seedlings germinated from brown seed. * means significant differences (*p* < 0.05) in IS vs. DS or S vs. IS and **#** means significant differences (*p* < 0.05) in S vs. DS. The abbreviations of clock gene names were listed with parentheses

Circadian regulation of hormone levels and hormonal signaling modulates many features of plant development, including seed dormancy and germination [26–28]. In this study, KEGG enrichment revealed that 23 DEGs (28 transcripts) were involved in circadian rhythm-plant pathway during black seed germination (Additional file 7: Table S6, Additional file 11: Table S10). As shown in Fig. 4, gene *COP1* (Unigene33753, Unigene33751), *CHS* (Unigene61566, Unigene5107, Unigene48137, Unigene19255), *TOC1* (Unigene45052), and *PIF7* (Unigene40139) displayed similar expression patterns in black seed, their expressions were kept at low levels at DS and IS stage, and increased markedly at S stage. On the contrary, gene *SPA2* (Unigene31560), *GI* (Unigene26154, Unigene26153), *SPA1* (Unigene22833, Unigene22832), *ZTL* (Unigene30382), *PHYA* (Unigene25752), *PRR9* (Unigene25684), and *FKF1* (Unigene22119, Unigene22120) were dramatically down-regulated at IS stage comparing to DS stage, and their expression were maintained at low levels at IS and S stage. The low expression levels of most of the clock genes at IS stage was possibly related to the dormancy-breaking and germination requirements of black seed. By contrast, in brown seed, only six out of 23 clock genes were developmentally regulated. Gene *PIF7* (Unigene40139) and *CHS* (Unigene5107, Unigene48137) were significantly up-regulated, *FKF1* (Unigene22120, Unigene22119) and *CHS* (Unigene8262) were markedly down-regulated at S stage when comparing to the other two stages. No statistically significant difference in the expression of clock genes was observed between DS and IS stage in brown seed.

### Small RNA sequence (sRNA-seq) analysis

**Fig. 5:**
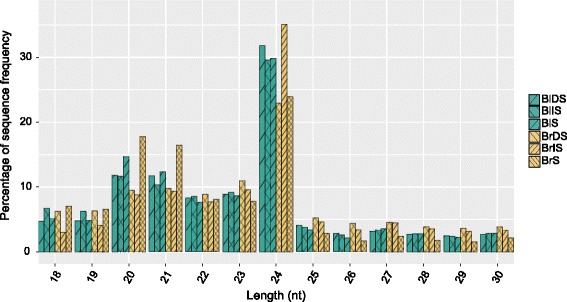
Length distribution of small RNA sequences in dry seed, imbibed seed, and seedling three libraries. BlDS represents black dry seed, BlIS represents black imbibed seed, BlS represents seedlings germinated from black seed, BrDS represents brown dry seed, BrIS represents brown imbibed seed, BrS represents seedlings germinated from brown seed. nt means nucleotides

Although black seed and brown seed share the same set of genes, they are different in many ways, including morphology, dormancy and germination characteristics [3]. These differences may be attributable to the genetic and epigenetic regulations, and in this study we focused on the developmental role of miRNA in *S. aralocaspica* germination. sRNA-seq generated 5,469,210 to 6,882,624 raw reads, after removing sequences shorter than 18 nt and greater than 30 nt in length, reliable clean reads ranging from 2,483,312 to 3,787,572 were collected for further analysis (Additional file 2: Table S11). The redundant clean reads were mapped to *S. aralocaspica* mRNA transcriptome database, 43.05% to 56.32% redundant sRNAs perfectly matched the *S. aralocaspica* transcript sequences. The high matching rate may be attributed to insufficient small RNAs. The majority of total sRNA reads ranged from 20 to 24 nt in length (Fig. 5), which are the typical size range of sRNAs generated by Dicer [29]. The major size of sRNA was 24 nt in all six libraries, the second most abundant class was 20 nt in the three libraries of black seed and BrS library, and 23 nt in the libraries of BrDS and BrIS.

### Identification of conserved and novel miRNA

**Fig. 6:**
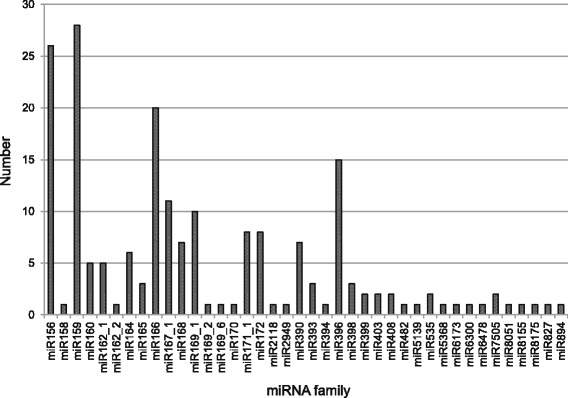
The number of members in each conserved miRNA family in *Suaeda aralocaspica*

A total of 194 conserved miRNAs were identified comprising 40 miRNA families (Additional file 12: Table S12), 21 nt and 20 nt were the two major size classes of conserved miRNAs. Among the identified conserved miRNA families, sar-miR156 and sar-miR159 were the two largest families that contained 26 and 28 members, respectively. Whereas, there were 19 miRNA families possessed only one member. The number of members for each family was summarized in Fig. 6. For precursor prediction, the transcript sequences of *S. aralocaspica* were used to determine hairpin structures. Four conserved miRNA precursors with lengths ranging from 87 nt to 171 nt were identified. Their minimal folding free energy indices (MFEIs) varied from 0.88 to 1.28 with an average of 1.09, which was consistent with that revealed in other plant miRNAs [30]. The total counts of conserved miRNAs were lowest in BlIS library and were rapidly increased in BlS library. In contrast, the total counts of conserved miRNAs were slightly increased in BrIS library compared to BrDS and reached the highest in BrS library (Additional file 12: Table S12). This finding indicated that the expression of conserved miRNAs in black seed was suppressed at IS stage.

We identified 22 putative novel miRNAs belonging to 20 families in *S. aralocaspica* and named them as sar-miR1 to sar-miR20 (Additional file 13: Table S13). Among the identified novel miRNAs, sar-miR13 was found to be homologous to sar-miR159 family members with one mismatch, therefore sar-miR13 was classified as a member of miR159 family. Sar-miR1a and sar-miR1b shared similar mature sequence, and sar-miR6a and sar-miR6b were homologous with each other, thereafter sar-miR1a and sar-miR1b were classified into sar-miR1 family, and sar-miR6a and sar-miR6b were classified into sar-miR6 family. The length of mature sequences of the novel miRNAs varied from 18 nt to 24 nt, and 21 nt was the major size class. By counting reads mapped to the different novel miRNAs in each sample, we found most novel miRNAs had relatively low expression levels, which was consistent with the feature of species-specific miRNAs. The length of novel miRNA precursors ranged from 61 nt to 170 nt, and MFEIs varied from 0.52 to 1.77 with an average value of 1.03. The total counts of novel miRNAs displayed a change trend similar to that of conserved miRNAs (Additional file 13: Table S13), suggesting that the expression of novel miRNAs in black seed was also suppressed at IS stage.

The precursor sequences and secondary hairpin structures of *S. aralocaspica* conserved and novel miRNAs predicted by Mfold [31] were represented in Additional file 14: Table S14 and Additional file 15: Figure S4.

### miRNA expression profiles

There were 49 miRNAs in black seed and 48 miRNAs in brown seed differentially expressed (DE) during dimorphic seed germination (*p*-value <0.01 and |log_2_FC| ≥1) (Fig. 1A, Additional file 16: Table S15). In the comparison of miRNA expression profiles between the three consecutive stages, we found that the numbers of DE miRNAs were dramatically increased in both seed at the phase transition from IS to S comparing to those at the phase transition from DS to IS, and the number of up-regulated miRNAs at the phase transition from IS to S in brown seed was much larger than that in black seed (Fig. 1B). Hierarchical cluster analysis illustrated that the expression profiles of DE miRNAs at DS and IS stages were similar for both seeds, DE miRNAs at S stage displayed a distinct expression pattern (Fig. 1C). These findings suggested that DE miRNAs majorly functioned in the phase transition from IS to S, and a larger number of miRNAs in brown seed took part in this transition process than black seed.

Based on their expression patterns, DE miRNAs were divided into four categories (Table 1). The first category contained miRNAs that were up-regulated during seed germination. The second category comprised miRNAs that were down-regulated during seed germination. The third category was composed of miRNAs that had relative low levels in dry seed, were up-regulated in imbibed seed then down-regulated in seedling. The last category contained miRNAs that were down-regulated in IS vs. DS, then up-regulated in S vs. IS. Among DE miRNAs, 43 conserved and nine novel miRNAs showed distinct expression patterns between black and brown seed during germination.

**Table 1:** Differentially expressed *Suaeda aralocaspica* miRNAs during seed germination

Black seed	Brown seed
Up-regulated	
sar-miR156t-5p, sar-miR159k, sar-miR160a, sar-miR164b, sar-miR166g, sar-miR168d, sar-miR169a, sar-miR169i, sar-miR171c, sar-miR172a, sar-miR172h, sar-miR393b, sar-miR396a, sar-miR396d, sar-miR396e, sar-miR396j, sar-miR396k, sar-miR396o, sar-miR6300a, sar-miR6b, sar-miR10	sar-miR319d, sar-miR160a, sar-miR164a, sar-miR164b, sar-miR166g, sar-miR167e, sar-miR169a, sar-miR171a, sar-miR171c, sar-miR172a, sar-miR172h, sar-miR390b, sar-miR396a, sar-miR396d, sar-miR396e, sar-miR396f, sar-miR396h, sar-miR396i, sar-miR396j, sar-miR396k, sar-miR396m, sar-miR396o, sar-miR398c, sar-miR6a, sar-miR10
Down-regulated	
sar-miR157a, sar-miR319d, sar-miR319h, sar-miR166b-3p, sar-miR166d, sar-miR166l, sar-miR166r, sar-miR167b, sar-miR167c, sar-miR167d, sar-miR167e, sar-miR167f, sar-miR167h, sar-miR167i, sar-miR167j, sar-miR171e-3p, sar-miR172b, sar-miR172c, sar-miR172e, sar-miR398c, sar-miR894a, sar-miR2, sar-miR7, sar-miR11, sar-miR12, sar-miR14, sar-miR16	sar-miR156n, sar-miR162b, sar-miR169d, sar-miR171h, sar-miR172b, sar-miR172c, sar-miR5139a, sar-miR5368a, sar-miR6173a, sar-miR6478a, sar-miR8155a, sar-miR8175a, sar-miR894a, sar-miR7, sar-miR11, sar-miR14, sar-miR17, sar-miR18, sar-miR19
Up-regulated then down-regulated	
sar-miR5	sar-miR159a, sar-miR159j
Down-regulated then up-regulated	
	sar-miR6300a, sar-miR5

### Target prediction of conserved and novel miRNAs

**Fig. 7:**
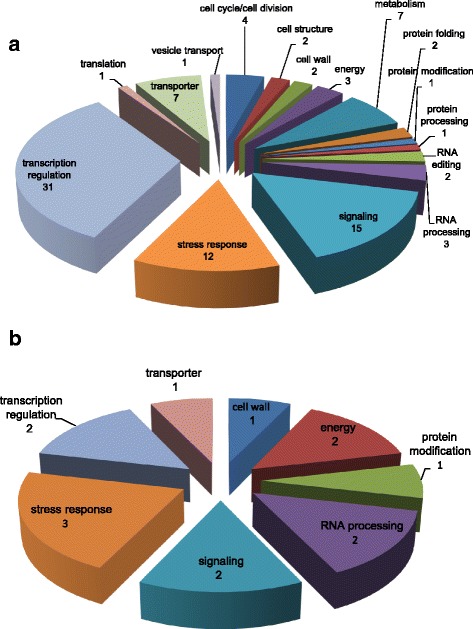
Functional classifications of predicted targets of conserved (**a**) and novel (**b**) miRNAs in *Suaeda aralocaspica*. Only the functionally annotated target genes are shown. The number of targets in each category is shown under the iterm

The 79,414 assembled transcripts from *S. aralocaspica* mRNA transcriptome database were used as a custom target database, 194 conserved and 22 novel mature miRNAs were used as a custom miRNA database. Using TAPIR [32], a total of 170 unigenes were predicted as potential targets of 35 conserved miRNA families (Additional file 17: Table S16). Thirty-three unigenes (19.41%) were homologous to the previously confirmed or predicted targets of the same miRNA families in *Arabidopsis thaliana*, *Oryza sativa* or *Solanum lycopersicum* (Table 2). Most of these conserved targets (23 out of 33) encoded essential transcription factors, and the rest of targets were homologs of Dicer protein, F-box protein, copper/zinc superoxide dismutase, inorganic phosphate transporter, plantacyanin, laccase, and CC-NBS-LRR protein. There were 137 putative targets of conserved miRNAs were not conserved in other plant species. Of these target genes, 76 (44.7%) targets presented no function annotation. The annotated 94 unigenes were classified into 16 categorizes according to their molecular and biological functions (Fig. 7A). Genes involved in transcription regulation (31, 33%; including 26 transcription factors) comprised the most dominant category, followed by unigenes involved in the other two main categories, signaling (15, 16%) and stress response (12, 13%). In the same way, 42 unigenes were identified as putative targets of 17 novel miRNA families and one miR159 family member (Additional file 18: Table S17). Most of these target genes (28, 66.67%) were not functionally annotated. The rest annotated targets were involved in eight categories (Fig. 7B). Unigenes involved in stress response (3, 22%), signaling (2, 14%), transcription regulation (2, 14%), energy (2, 14%), and RNA processing (2, 14%) accounted for the major proportions.

**Table 2:** Conserved^a^ miRNA targets identified in *Suaeda aralocaspica*

miRNA family	Target	Annotation
miR156/157	Unigene25708	squamosa promoter-binding-like protein 6 isoform X2
	Unigene30985	squamosa promoter-binding-like protein 2
miR159	Unigene5343	MYB protein, DUO POLLEN 1
miR160	Unigene37068	auxin response factor 10
	Unigene36784	auxin response factor 16
	Unigene39132	auxin response factor 17
miR162	Unigene29941	SUSPENSOR 1, Dicer-like 1
miR164	Unigene38871	NAC domain containing protein 100
miR165/166	Unigene32963	member of HD-ZIP III family, INCURVATA 4
	Unigene47107	member of HD-ZIP III family, REVOLUTA
miR169	Unigene44960	CCAAT binding factor-HAP2-like protein, nuclear factor Y subunit A1
	Unigene26545	CCAAT binding factor-HAP2-like protein, nuclear factor Y subunit A9
	Unigene25577	nuclear factor Y subunit A-3
miR170/171	Unigene34442	HAIRY MERISTEM 3, SCL6-IV
	Unigene44608	HAIRY MERISTEM 4, SCL15
miR172	Unigene26235	TARGET OF EARLY ACTIVATION TAGGED 1
	Unigene13804	AP2-like ethylene-responsive transcription factor TOE3
miR319	Unigene42701	TEOSINTE BRANCHED 1, cycloidea and PCF transcription factor 2
	Unigene46584	TEOSINTE BRANCHED 1, cycloidea and PCF transcription factor 3
miR393	Unigene37071	F-box protein, TRANSPORT INHIBITOR RESPONSE 1
	Unigene37070	F-box protein, TRANSPORT INHIBITOR RESPONSE 1
	Unigene47621	Auxin signaling F-box 3
miR394	Unigene26101	a putative F-box protein, LEAF CURLING RESPONSIVENESS
miR396	Unigene44576	growth-regulating factor 2
	Unigene20645	growth-regulating factor 3
	Unigene24713	growth-regulating factor 4
	Unigene44047	growth-regulating factor 5
	Unigene20255	growth-regulating factor 7
miR398	Unigene2632	copper/zinc superoxide dismutase 1
miR399	Unigene40813	a ubiquitin-conjugating E2 enzyme, UBIQUITIN-CONJUGATING ENZYME 24
miR408	Unigene47976	plantacyanin
	Unigene39882	laccase 3
miR482	Unigene39969	disease resistance protein, CC-NBS-LRR class

^a^Conserved with *Arabidopsis thaliana*, *Oryza sativa*, or *Solanum lycopersicum*

Due to insufficient *S. aralocaspica* mRNA sequences, we were not able to predict the targets for five conserved miRNA families and two novel miRNA families.

### Validation of miRNA targets by 5′ rapid amplification of cDNA ends (RACE)

**Fig. 8:**
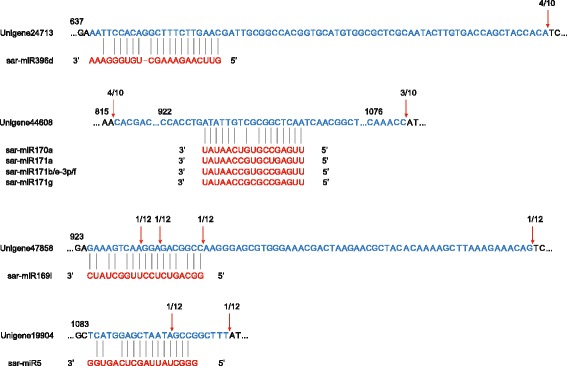
Validation of miRNA-guided target unigene cleavage. Partial sequences from target genes were aligned with the corresponding miRNAs. Each top strand (blue) represents a miRNA-homologous site in the target gene and each bottom strand (red) represents the aligned sequence of miRNA. Red arrows indicate the observed miRNA cleavage sites following 5′ RACE analysis, with the frequency of clones shown

To validate the results of *in silicon* analysis, we amplified the predicted target genes through 5′ RACE-PCR. The cleavage sites in four predicted target genes of *S. aralocaspica* were successfully detected (Fig. 8). Unigene24713, Unigene44608, Unigene47858, and Unigene19904 were confirmed to be targets of sar-miR396d, sar-miR170/171, sar-miR169i, and sar-miR5, respectively. Sequencing of the sar-miR169i-cleaved 5′ RACE product of Unigene47858 identified a precise slice between the nucleotides 10 and 11 in the complementary region of the miRNA: mRNA pair (Fig. 8). Unigene47858 encodes a protein homologous to the Arabidopsis late embryogenesis abundant (LEA) protein family protein. Unigene44608 and Unigene19904 were validated to be targets of sar-miR170/171 and sar-miR5, respectively, with multiple cleavage sites. Unigene44608 is homologous to an Arabidopsis protein coded by GRAS family transcription factor, and Unigene19904 is homologous to an Arabidopsis protein coded by protein kinase superfamily protein. Unigene24713, the putative target of sar-miR396d, was also evaluated for its cleavage site. Unigene24713 encodes a protein homologous to the Arabidopsis transcription factor growth-regulating factor 4. A shorter or longer cleaved sequence was observed for the four putative targets after 5′ RACE analysis, which could be attributed to secondary siRNA in the 21-nucleotide register with the cleavage site for miRNAs as previously documented [33–35].

### Validation of the expression profiles of unigenes and miRNAs by real-time quantitative reverse transcription (qRT)-PCR

To validate the expression of identified unigenes and miRNAs from transcriptome analysis, six GA or ABA signal genes (Unigene20723, Unigene49059, Unigene2681, Unigene3190, Unigene28427, and Unigene38625), five conserved miRNAs (sar-miR166l, sar-miR166r, sar-miR169i, sar-miR394a and sar-miR396e) and their potential targets (Unigene32963, Unigene47858, Unigene26101, Unigene44047), and three novel miRNAs (sar-miR5, sar-miR7 and sar-miR17) were selected and subjected to the qRT-PCR analysis. For calculating the relative expression of each unigene or miRNA, the Ct value at DS stage was used as a reference. Analysis of transcript levels by qRT-PCR showed that the ten unigenes (*R* ^2^ = 0.459, *P* < 0.01) and eight miRNAs (*R* ^2^ = 0.402, *P* < 0.01) all displayed positive correlation between the RNA/sRNA-seq quantitative measurement and qRT-PCR method (Additional file 19: Figure S5, Additional file 20: Table S18), suggesting that most of the tested unigenes and miRNAs showed similar developmental alterations in the two methods and our high-throughput data were reliable.

Further, we assessed the expression patterns of five miRNAs and their putative targets using real-time qRT-PCR. As shown in Additional file 21: Figure S6, the expression of sar-miR166l and sar-miR166r was suppressed while the level of Unigene32963 was increased during black seed germination. The level of sar-miR169i was enhanced while the expression of Unigene47858 was inhibited at BrS stage. The expression of sar-miR394a was decreased, whereas, the level of Unigene26101 was elevated at BlS stage. The level of sar-miR396e was increased while the expression of Unigene44047 was repressed at both BlS and BrS. The putative target genes exhibited opposite expression patterns to their corresponding miRNAs at certain time points of germination, suggesting that these targets may be regulated post-transcriptionally by the action of miRNAs.

## Discussion

This study provided an overview of the genes and miRNAs presented in a non-model euhalophyte species and identified the candidates associated with the germination process of dimorphic seed under the control of a bet-hedging strategy.

### The metabolic processes activated earlier in brown seed than in black seed during *S. aralocaspica* seed germination

It is documented that mature seeds possess photo-synthetically active chloroplasts, which maintain photosynthetic activity during the period of reserve accumulation, contributing oxygen supply and coupled biosynthetic fluxes [36–38]. Morphologically, the membranous seed coat of *S. aralocaspica* brown seed (Additional file 1: Figure S1A) would allow the embryo gain more amount of light than the black seed embryo, which is regarded as a critical factor for seed photosynthesis. In order to comprehensively understand the initial important metabolic processes activated at the transition from dry seed to germination, we combined the KEGG enrichment results of DEGs in dry seeds and during seed germination. We found that some KEGG pathways were activated in both seed germination, but they were activated earlier in brown seed compared with black seed. For instance, “Photosynthesis-antenna proteins”, “Photosynthesis”, and “Flavone and flavonol biosynthesis” pathways were activated at DS stage in brown seed (Fig. 2B, Additional file 9: Table S8), but activated at IS and S stages in black seed (Fig. 2A, Additional file 7: Table S6). Among 12 antenna proteins and 17 photosynthesis proteins that were up-regulated at DS stage in brown seed compared with black seed, 12 antenna proteins and 15 photosynthesis proteins were up-regulated at the late stages of germination in black seed (Fig. 2, Additional file 22: Table S19, Additional file 23: Table S20). This finding provided strong evidence that photosynthesis process was activated earlier in brown seed than black seed during *S. aralocaspica* germination. Flavonols have been shown to negatively regulate auxin transport and dependent physiological processes [39–43]. Auxin gradient is crucial for de novo induction and maintenance of root meristematic activity [44–46]. Flavonol biosynthesis is induced by auxin [42], and the expression of genes involved in this pathway is light dependent [47, 48]. In *S. aralocaspica*, the activation of flavonol biosynthesis at DS stage in brown seed demonstrated that seedling establishment could be initiated earlier in brown seed than in black seed. Additionally, miR393 targets F-box genes that encode auxin receptors [49–51], and TRANSPORT INHIBITOR RESPONSE 1 (TIR1) acts as an auxin receptor mediating transcriptional responses to auxin [52]. In this study, Unigene37070 and Unigene37071, encoding proteins homologous to TIR1, were predicted as targets of sar-miR393b (Table 2, Additional file 17: Table S16). sar-miR393b was up-regulated at S stage comparing to the other two stages in black seed (Table 1, Additional file 16: Table S15), while maintained at a high level in brown seed. Unigene37070 and Unigene37071 showed opposite expression patterns to sar-miR393b (Additional file 5: Table S4) during germination. These findings suggested that the distinct expression patterns of miR393 and its targets between black and brown seed maybe associated with the early activation of flavonol biosynthesis in brown seed.

### Candidate ABA and GA genes that may contribute to the diversity in seed germination behaviors in *S. aralocaspica*

Protein phosphatase 2CA (PP2CA) is generally up-regulated in response to ABA in *Thellungiella salsuginea* [53] and salt treatment in *Suaeda fruticosa* [54]. In *S. aralocaspica*, the expression of *PP2CA* (Unigene3190) was gradually increased in black seed but continuously decreased in brown seed (Fig. 3, Additional file 10: Table S9), suggesting that PP2CA may play a role in stress response during black seed germination. In Arabidopsis, three subclass III SNF1-related kinase 2 s (SnRK2s), SRK2D/SnRK2.2, SRK2E/SnRK2.6/OST1 and SRK2I/SnRK2.3 (SRK2D/E/I), are strongly activated by ABA and osmotic stresses [55, 56]. At present study, one homologous of OST1 (Unigene28427) was up-regulated in black seed but maintained at a relative high level in brown seed (Fig. 3, Additional file 10: Table S9), implying that OST1 may be activated earlier in brown seed than black seed, and it could be involved in ABA and osmotic stress response in both seed during *S. aralocaspica* germination process. One homologous of SnRK2.3 (Unigene14586) was only detected at S stage in brown seed (Fig. 3, Additional file 10: Table S9), suggesting this gene may play a role in stress tolerance in brown seed during post-germination growth. GA promotes seed germination by enhancing the proteasomal destruction of ral guanine nucleotide dissociation stimulator-like 2 (RGL2) [57], and F-box protein SLEEPY1 (SLY1) is required for this process [58]. In this study, SLY1 showed different expression trend between black and brown seed (Fig. 3, Additional file 10: Table S9), indicating that GA signaling maybe regulated differently during dormant and non-dormant seed germination. Further, there were several receptors and downstream transcription factors of ABA or GA signaling, including PYL2 (Unigene40508), PYL12 (Unigene2681), AREB3 (Unigene38625 and Unigene40984), and GID1C (Unigene20723), showed distinct expression patterns between black and brown seed during germination (Fig. 3, Additional file 10: Table S9). This difference may contribute to the different germination behaviors of *S. aralocaspica* dimorphic seeds in order to cope with the harsh and unpredictable environment. Further investigation on the molecular mechanisms underlying the dimorphic seed germination controlled by GA and ABA signaling is needed.

### Candidate clock genes that may contribute to dormancy breaking during black seed germination

Using real-time RT-PCR, Arabidopsis circadian clock has been demonstrated to act as an important signal integrator regulating dormancy release [26]. In current study, the high throughput data allowed us to comprehensively compare the dynamic expression changes occurred in dormant seed and non-dormant seed during germination in the same species. KEGG enrichment identified 23 clock genes participated in the circadian rhythm pathway in *S. aralocaspica* black seed (Additional file 11: Table S10). These 23 genes exhibited the well-characterized transcriptional circadian rhythms of dormancy breaking. The transcript levels of *SPA*, *GI*, *ZTL*, *PHYA*, *PRR9*, and *FKF1* were high and the transcript levels of *COP1*, *CHS*, *TOC1*, and *PIF7* were relatively low in non-deep dormant dry seed (Fig. 4). At IS stage, seed imbibition and testa rupture had a general suppression effect on the gene expressions of *GI*, *CHS*, *SPA*, *ZTL*, *PHYA*, *PRR9*, and *FKF1*, indicating the clock components in imbibed black seeds may be responding to the environmental and endogenous signals for dormancy-breaking requirement. At S stage, uncoiled embryo rupture promoted the expressions of *GI*, *COP1*, *CHS*, *TOC1*, and *PIF7*, which possibly initiated the seedling establishment of circadian oscillations in black seed. In contrast, brown seed exhibited a different transcriptional profile of these 23 clock genes and the circadian rhythms seemed to persist during the whole germination process, which was consist with its genotype that seedling establishment-related biological process initiated early at dry seed stage (Fig. 2B, Additional file 9: Table S8).

### The transcriptional suppression of miRNAs at IS stage in black seed

Similar to the transcriptional suppression of clock genes at BlIS stage, we found the total counts of conserved and novel miRNAs were lowest at IS stage in black seed comparing to the other two stages (Additional file 13: Table S13, Additional file 14: Table S14), implying that the expression levels of miRNAs could be suppressed by dormancy breaking either. We speculated that transcriptional suppression maybe one crucial success factor for non-deep dormant seed to break dormancy. In plants, DICER-LIKE 1 (DCL1) is the main processor in miRNA biogenesis [59] and subject to negative feedback regulation through miR162 [60]. At present study, we predicted that sar-miR162b targeted one DCL1 protein (Table 2, Additional file 17: Table S16). In black seed, the expression of sar-miR162b was kept at the same level at DS and IS, then was decreased at S stage (Table 1, Additional file 16: Table S15). In brown seed, the expression level of sar-miR162b was relatively high at DS, then decreased at IS and further decreased at S. This finding indicated that miR162 could play an important role in transcriptional suppression of miRNAs and dormancy breaking through its target DCL1.

### miRNAs with distinct expression patterns between the dimorphic seeds and their roles in *S. aralocaspica* seed germination

The germination process is a coordinated action of multiple environmental responsive genes, which also cross-talk with other components of the elaborate hormone signaling networks. miRNAs play critical roles in the regulation of these biological processes [61, 62]. Arabidopsis miR156 is essential for vegetative leaf development from the cotyledon-stage seedlings by down-regulating its target SPL [63, 64]. Maize miR156 is differentially down-regulated at imbibition step during seed germination [65]. In this study, sar-miR156t-5p was up-regulated in black seed during germination (Table 1, Additional file 16: Table S15), whereas, no significant sar-miR156t-5p expression difference was identified between the germination stages in brown seed. Two SPL proteins were predicted as the targets of sar-miR156t-5p (Table 2, Additional file 17: Table S16), implying miR156 could play important roles in vegetative leaf development from black seed seedling by regulating SPL, while brown seed may depend less on this mechanism in vegetative leaf development after germination. miR159 is induced by ABA, suppresses MYB33 and MYB101 transcript levels and renders plants hyposensitive to ABA during germination [66]. miR159 was also induced under drought conditions suggesting it maybe a signal factor in sensing the environment surrounding a seed to ensure plant survival after germination [62, 66]. At present study, sar-miR159k was up-regulated in black seed, while sar-miR159j were up-regulated in IS vs. DS but down-regulated in S vs. IS in brown seed during germination (Table 1, Additional file 16: Table S15). One MYB protein was predicted to be the target of sar-miR159j and sar-miR159k (Table 2, Additional file 17: Table S16), indicating that miR159-mediated regulatory module may be linked with ABA responses and homeostasis during *S. aralocaspica* seed germination, and desensitize hormone signaling to tolerate adverse environments in black seed during post-germination growth. SUPPRESSOR OF MAX2 1 (SMAX1) is an important component of karrikins (KAR) / strigolactone (SL) signaling, regulating germination and hypocotyl elongation [67]. In this study, we predicted that sar-miR167e targeted a homolog of SMAX1 (Additional file 17: Table S16). sar-miR167e was down-regulated in black seed but up-regulated in brown seed during germination (Table 1, Additional file 16: Table S15), suggesting miR167 may regulate germination and hypocotyl elongation in different ways between black and brown seed. miR171-targeted scarecrow-like (SCL) 6/22/27 proteins mediate GA-DELLA signaling in the coordinate regulation of chlorophyll biosynthesis under light conditions [68]. SCL15 plays an essential role in repressing embryonic traits in Arabidopsis seedlings [69]. At present study, sar-miR171e-3p was down-regulated in black seed in IS vs. DS, and sar-miR171a was up-regulated and sar-miR171h was down-regulated in brown seed during germination (Table 1, Additional file 16: Table S15). A homolog of SCL6-IV was predicted as the target of sar-miR171a/e-3p/h, and a homolog of protein of SCL15 was predicted to be the target of sar-miR171a/e-3p (Table 2, Additional file 17: Table S16), implying that miR171-SCL module may fine-tune the GA-regulated chlorophyll biosynthesis and participate in the regulation of embryo-to-seedling phase transition during *S. aralocaspica* seed germination.

miRNAs also play an important role in various stress responses. miR169 is induced by drought [70] and high salinity [71] in rice, but repressed by salt in *Thellungiella salsuginea* [72]. miR169 targeted *NF-YA* encodes a subunit of the NF-Y complex transcription factor which is involved in root development, nitrogen-starvation responses, and plant responses to drought and salt stresses [71, 73, 74]. LEA proteins have crucial roles in cellular dehydration tolerance [75]. At present study, sar-miR169i was up-regulated in black seed, while sar-miR169d was down-regulated in brown seed during germination (Table 1, Additional file 16: Table S15). We predicted that sar-miR169d targeted one NF-YA transcription factor and sar-miR169i targeted one LEA protein (Table 2, Additional file 17: Table S16), indicating that miR169 may exert diverse roles in response to drought and salt stresses during *S. aralocaspica* germination. Phosphatase 2C (PP2C) family proteins are key players in ABA signal transduction during seed germination [24, 76]. Highly ABA-induced PP2C gene 2 (HAI2) is a member of the PP2C family and recently found to be also involved in ABA-independent drought-associated signaling [77]. One FK506-Binding Protein (FKBP) family protein, ROF1, is reported to play an important role in salt stress responses during Arabidopsis seed germination [78]. In this study, sar-miR172e was down-regulated in black seed during germination (Table 1, Additional file 16: Table S15), whereas, no significant difference in sar-miR172e expression was identified between the germination stages in brown seed. A homolog of HAI2 and one FKBP protein were predicted to be the targets of sar-miR172e (Additional file 17: Table S16), indicating that miR172 may participate in ABA-dependent and ABA-independent stress signaling during black seed germination through its targets. miR398 expression is induced by salt treatment in Populus [79] and *T. salsuginea* [72], but is repressed by oxidative stresses in Arabidopsis [80] and high levels of copper and cadmium in *Medicago truncatula* [81]. The targets of miR398 are Cu/Zn superoxide dismutase (CSD) that can detoxify superoxide molecules. When copper supply is limited, the accumulation of miR398 reduces the allocation of copper into CSDs and saves copper for other essential processes [82, 83]. In *S. aralocaspica*, sar-miR398c was down-regulated in black seed while up-regulated in brown seed at S stage when comparing with the other two stages (Table 1, Additional file 16: Table S15). Although no unigene was predicted as the target of sar-miR398c in this study, we assumed that miR398 may play an important role in mediating the copper homeostasis that is required for photosynthetic and respiratory electron transport, oxidative stress protection, cell wall metabolism [84] during black and brown seed germination. Kinases and phosphatases have been documented to be involved in the regulation of proteins involved in osmolyte synthesis and detoxification by oxidants [54, 85]. They may play a role in salinity tolerance. In this study, a protein kinase superfamily protein was the candidate target of sar-miR5 (Additional file 18: Table S17). sar-miR5 was up-regulated in IS vs. DS and down-regulated in S vs. IS in black seed, but was down-regulated in IS vs. DS and up-regulated in S vs. IS in brown seed (Table 1, Additional file 16: Table S15), suggesting sar-miR5 may regulate salt tolerance through its target in diverse pathways in black and brown seed during *S. aralocaspica* germination. Wall associated kinase-like (WAKL) 1 and putative indole-3-acetic acid (IAA)-amido synthetase GH3.9 were predicted to be the targets of sar-miR18 (Additional file 18: Table S17). WAKL members respond to environmental stresses and are developmentally regulated and tissue specific [86]. In Arabidopsis, WAKL1 has highest expression level in roots [86]. GH3.9 functions as IAA-amido synthetase to conjugate amino acids to the plant hormone auxin. gh3.9–1 mutants had greater primary root length, and increased sensitivity to IAA-mediated root growth inhibition [87]. At present study, sar-miR18 was down-regulated at S stage comparing to DS stage in brown seed (Table 1, Additional file 16: Table S15), while no significant sar-miR18 expression difference was identified between the germination stages in black seed, implying sar-miR18 maybe involved in environmental stresses response and root growth during germination and early seedling growth of brown seed by regulating *WAKL1* and *GH3.9* genes.

### Candidate miRNAs that may be related to the cautious germination strategy of black seed

In plants, stem cells positioned in shoot apical meristem (SAM) and root apical meristem (RAM) constitute a pool of undifferentiated cells that continually provides new cells for post-embryonic growth [88]. miR166/165 have a conserved role in the maintenance of shoot and root apical meristems activity by negatively regulating its target, CLASS III HOMEODOMAIN-LEUCINE ZIPPER (HD-ZIP III) [89–91]. In *S. aralocaspica*, sar-miR166b-3p/d/l/r were down-regulated in black seed at S stage comparing to the other two stages (Table 1, Additional file 16: Table S15), while maintained at a high level in brown seed. Three HD-ZIP III transcription factors were predicted as the targets of sar-miR166b-3p/l/r, and one HD-ZIP III transcription factor was predicted to be the target of sar-miR166d (Table 2, Additional file 17: Table S16). This finding indicated that black seed may have a lower meristem activity than brown seed at seedling stage. The down-regulation of sar-miR166b-3p/d/l/r at S stage could be a cautious germination strategy for black seed to respond quickly and proactively to the precarious environment with low risk to seedling survival.

## Conclusions

In this study, we performed a systematic analysis of genes and miRNAs in *S. aralocaspica*. Our data revealed that specific genes and miRNAs were regulated differently between black and brown seed during germination. These candidate genes/miRNAs may contribute to the different germination behaviors of *S. aralocaspica* dimorphic seeds under the control of a bet-hedging strategy. This study elucidated the molecular mechanisms underlying the control of the timing of *S. aralocaspica* germination, stress tolerance during dimorphic seed germination, and the cautious germination strategy of black seed. The findings of this study provided a solid foundation for further understanding of the heteromorphic seed germination of halophytes in desert regions.

## Methods

### Plant materials

Freshly matured fruits of *Suaeda aralocaspica* were collected from plants in a natural population (44^o^14’ N; 87^o^44’ E; 445 m a.s.l) growing at the Fukang Desert Ecosystem Observation and Experimental Station in Xinjiang Province, China in early October 2013. The specimens used in this study were not deposited in a herbarium. Fruits were dried naturally for ten days under ambient room conditions. After that, seeds were separated from the dried plant material and sorted into black and brown seeds. *S. aralocaspica* black and brown dry seeds were sown on two layers of Whatman paper soaked with distilled water and incubated in a cabinet at 25 °C with continuous light. Imbibed brown seed were harvested 1 h after sowing, seedlings from brown seeds were harvested 24 h after sowing. Imbibed black seed were harvested 24 h after sowing, seedlings from black seeds were harvested within 10 d after sowing. Collected samples were frozen in liquid nitrogen and stored at −80 °C for further analysis.

### cDNA library preparation and RNA-seq

Total RNA was extracted by using TRIzol® reagent (Invitrogen, Carlsbad, CA, USA) according to the manufacturer’s instructions, and RQ1 DNase (Promega, Madison, WI, USA) was used to remove contaminating genomic DNA. The quality and quantity of the purified RNA was monitored at the ratios of A260/A280 and A260/230 on SmartSpec Plus Spectrophotometer (BioRad, Philadelphia, PA, USA). RNA integrity was further verified by 1.5% agarose gel electrophoresis and assessed by Agilent 2100 Bioanalyzer (Agilent Technologies, Santa Clara, CA, USA).

Equal amounts of RNA isolated from the samples collected at the same stages (DS, IS, S) were mixed together to prepare the cDNA library. mRNAs were purified and concentrated with Magnetic Beads Oligo (dT) (Invitrogen, Carlsbad, CA, USA). Purified mRNAs were iron fragmented at 95 °C followed by end repair and 5′ adaptor ligation. Then, reverse transcription was performed with RT primer harboring 3′ adaptor sequence and randomized hexamer. Six cDNA libraries with insert sizes from 300 to 500 bp were prepared for Illumina HiSeq 2000 system 101 nt pair-end sequencing.

### RNA-seq data filter

We removed the low-quality reads with these criteria: 1) raw reads containing more than 2-N bases were removed, 2) the reads were processed by clipping adaptor, 3) low quality bases were removed, 4) too short reads (less than 16 nt) were removed. FASTX-Toolkit [92] (Version 0.0.13) was used to filter the raw reads. All RNA sequencing reads were deposited to NCBI under BioProject accession number PRJNA325861.

### Assembly and statistics

We used Trinity [16] with a 25-mer parameter for de novo assembly of the clean reads to generate a non-redundant set of transcripts, other default parameters including: group_pairs_distance = 500, path_reinforcement_distance = 70, min_kmer_cov = 1. Afterwards, we realigned all clean reads onto the transcripts using Bowtie 2 [19] (Version 2.2.9), allowing up to four-base mismatches. We calculated the read coverage of each transcript. A transcript was defined not to be false positive, if the read coverage (the depth at least one read) was over 90% of the transcript.

After Trinity assembly, we used CD-HITv4.6.4–2015-0603 [20] for obtaining distinct sequences (transcripts). The following parameters were used to ensure quality of assembly: 1) sequence identity threshold: 0.95, 2) alignment coverage for the shorter sequence: 0.9, 3) maximum unmatched percentage (excluding leading and tailing gaps) for the shorter sequence must not be more than 10% of the sequence.

### Annotation and predicted CDS

All assembled transcripts were searched against Nr, COG, and Swiss-Prot protein database with BLASTX althorithm [93], and KEGG by BLAST2GO [21]. The E-value cut-off was set to 10^−5^. Genes were identified according to best hits against known sequence functions, prediction of GO terms were also performed. The CDS were selected from transcript sequences based on the above alignment results, and transcripts not uncovered in the results were predicted by ESTScan [94]. The shortest CDS were at least 100 bp.

In the meanwhile, transcripts were annotated by *B. vulgaris* genome [95] and *P. euphratica* genome [96] and their whole proteome using BLAT [97] and BLASTX [93] (E-value <10^−5^), respectively.

### Differential expression analysis of unigenes

All clean reads were realigned onto the assembled transcripts using Bowtie 2 [19], allowing up to four-base mismatches. Trinity is able to report all alternatively spliced isoforms and transcripts derived from paralogous genes [16]. In order to calculate the read number and RPKM value for each gene, the alternative isoforms or paralogous transcripts were merged as one gene, the reads aligned with more than one gene were discarded due to their ambiguous location. Uniquely localized reads were used to calculate read number and RPKM value for each gene. To determine the differentially expressed unigenes between any two germination stages of *S. aralocaspica*, gene expression level analysis was performed using EdgeR package [22]. For each gene, the *p*-value and FDR were obtained based on the model of negative binomial distribution. The fold change (FC) of expression was also calculated within EdgeR. |log_2_FC| ≥1 and *p*-value <0.01 were set as the threshold to define DEGs. Identified DEGs were further annotated using BLASTX [93] against Nr and Arabidopsis Information Resource (TAIR) database with a cut-off E-value of 10^−5^.

### sRNA library construction

Five to six samples collected at each germination stage were mixed together, total RNA was extracted from the mixture. Three μg of each RNA sample was used for sRNA cDNA library preparation with Balancer NGS Library Preparation Kit (GnomeGen, San Diego, CA, USA) based on manufacturer’s instruction. Whole library was applied to 10% native PAGE gel electrophoresis and bands corresponding to miRNA insertion were cut and eluted. After ethanol precipitation and washing, the purified small RNA libraries were quantified with Qubit Fluorometer (Invitrogen, Carlsbad, CA, USA) and used for cluster generation and applied to Illumina GAIIx (Illumina, San Diego, CA, USA) 73 nt and Illumina HiSeq 2000 100 nt single-end sequencing.

### Bioinformatic analysis of the sRNA transcriptome

All sequencing data was processed by FASTX-Toolkit [92], adaptor sequences and low quality tags were filtered. Based on the length of the mature miRNA and adaptor length, sequences shorter than 18 nt and greater than 30 nt in length were removed. At this step, the data were screened for redundant sequences. The remaining sequences were mapped to Rfam database (version 11.0 [98]) and *S. aralocaspica* mRNA transcriptome database for perfect matches, using custom-written PERL script. The matches to rRNAs, tRNAs or mRNAs were excluded. To identify conserved miRNAs, the retained unique sequences were aligned against miRBase (version 21), which contains 8582 miRNAs across 75 plant species [99], and the newly identified 241 miRNAs in *Salicornia europaea* [33], using Bowtie 2 [19] (one mismatch allowed). Only the perfectly matched sRNA sequences were considered to be conserved miRNAs. To reveal conserved and novel miRNA precursors, the unique sequences that have 10 or more counts were aligned to the *S. aralocaspica* mRNA transcriptome database using miRDeep-P with default parameters [100]. BLASTX [93] was used to match the sequences to Nr database, the putative precursors conserved in other plant species were removed. Mfold [31] was used to predict the secondary structures of the putative precursors utilizing default parameters. The miRNA precursors should met the following criteria: 1) forming an appropriate stem-loop structure, with a mature miRNA sitting in one arm of the hairpin structure; 2) mature miRNAs had no more than 6 mismatches with the opposite miRNA sequences; 3) the minimal folding free energy (MFE) of the hairpin structure was less than −15 kcal/mol; 4) MFE index were more than 0.5; 5) A + U content was between 30 and 70% [33, 101].

All small RNA sequencing reads were deposited to NCBI under BioProject accession number PRJNA325861.

### Differential expression analysis of miRNAs

All clean reads from sRNA libraries were aligned against the mature sequences of identified miRNAs (only one mismatch allowed), the mapped reads were normalized to tags per million (TPM). Differentially expressed miRNAs between the three stages, DS, IS, S, were analyzed using Fisher Exact Test [102], and the mapped reads were pre-normalized by the locally weighted scatter plot smoothing method. *p*-value <0.01 and |log_2_FC| ≥1 were set as the threshold to define DE miRNAs.

### miRNA target prediction and validation

The potential miRNA targets were identified using TAPIR [32] with default settings. All predicted target transcripts were evaluated by scoring system and considered to be miRNA targets if TAPIR score was less than 3.5 and miRNA-transcript duplex free energy ratio (mfe_ratio) was more than 0.7.

Two μg total RNA from equally mixed six RNA extractions of DS, IS, and S was used to synthesize 5′-RACE-ready cDNAs with the 5′-Full RACE Kit (Takara Bio Inc., Otsu, Shiga, Japan) according to the manufacturer’s instructions. The final PCR product was extracted and purified from a 2% agarose gel, cloned into pEASY-T1 Vector (Beijing TransGen Biotech Co., Ltd., Beijing, China), and plasmid DNA from ten to twelve different colonies was sequenced. The outer and inner gene specific primers were listed in Additional file 24: Table S21.

### qRT-PCR

One microgram of total RNA was reverse transcribed using M-MLV Reverse Transcriptase according to the manufacturer’s protocol (Promega, Madison, WI, USA). We selected 18 s rRNA and U6 snRNA as the endogenous controls. The primers for examined unigenes were designed by us from the *S. aralocaspica* transcriptome sequences and optimized for PCR (Additional file 24: Table S21), and bulge-loop qRT-PCR primers for mature miRNAs and U6 were designed and provided by RIBOBIO (Guangzhou RIBOBIO Co., Ltd., Guangzhou, China). Real-time monitoring of PCR was performed with ABI3700 (Applied Biosystems, Grand Island, NY, USA) and TransStart Top Green qPCR SuperMix (TransGen Biotech, Beijing, China). Reaction was performed at 95 °C for 10 min, and then cycled at 95 °C for 15 s, 60 °C for 60 s for 40 cycles. Each assay was performed in triplicate, real-time qRT-PCR data were analyzed based on 2^-ΔΔCt^ method [103].

## Additional files

Additional file 1: Figure S1. Morphology of seed germination in *Suaeda aralocaspica*. Bar = 1 mm. (PDF 13983 kb)

Additional file 2: Table S1. Statistics of RNA sequencing reads. **Table S2.** Summary of de novo sequence assembly using Trinity. **Table S3.** Annotation on assembled transcripts by different public databases. **Table S11.** Statistics of small RNA sequences from the six small RNA libraries. (DOCX 59 kb)

Additional file 3: Figure S2. GO functional classification of *Suaeda aralocaspica* unigenes within the category of cellular component (A), biological process (B) and molecular function (C). The number of unigenes enriched in each subcategory is indicated above each bar. (PDF 406 kb)

Additional file 4: Figure S3. Length distribution of *Suaeda aralocaspica* assembled transcripts and coding region sequences (CDS) of the transcripts. nt, nucleotides. (PDF 507 kb)

Additional file 5: Table S4. Unigene RPKM value and annotation against Nr database at NCBI. (XLSX 5600 kb)

Additional file 6: Table S5. Unigenes differentially expressed during *Suaeda aralocaspica* seed germination. (XLSX 3688 kb)

Additional file 7: Table S6. Annotation of unigenes differentially expressed during *Suaeda aralocaspica* seed germination compared with KEGG database. (XLSX 67 kb)

Additional file 8: Table S7. Unigenes differentially expressed between black and brown dry seed. (XLSX 270 kb)

Additional file 9: Table S8. Annotation of unigenes differentially expressed between black and brown dry seed compared with KEGG database. (XLSX 27 kb)

Additional file 10: Table S9. Identification of unigenes involved in abscisic acid and gibberellic acid signal transduction. (XLSX 17 kb)

Additional file 11: Table S10. Identification of differentially expressed unigenes involved in circadian rhythm-plant pathway during black seed germination. (XLSX 12 kb)

Additional file 12: Table S12. Identification of conserved miRNAs in *Suaeda aralocaspica*. (XLSX 76 kb)

Additional file 13: Table S13. Identification of novel miRNAs in *Suaeda aralocaspica*. (XLSX 35 kb)

Additional file 14: Table S14. The precursor sequences of *Suaeda aralocaspica* miRNAs. (XLSX 32 kb)

Additional file 15: Figure S4. The secondary structures of *Suaeda aralocaspica* miRNAs predicted by Mfold. The mature sequences of miRNA were highlighted in yellow. (PDF 2399 kb)

Additional file 16: Table S15. miRNAs differentially expressed during Suaeda aralocaspica seed germination. (XLSX 57 kb)

Additional file 17: Table S16. The putative targets of Suaeda aralocaspica conserved miRNAs predicted by TAPIR. (XLSX 49 kb)

Additional file 18: Table S17. The putative targets of Suaeda aralocaspica novel miRNAs predicted by TAPIR. (XLSX 41 kb)

Additional file 19: Figure S5. Validation of the expression profiles of selected unigenes (A) and miRNAs (B). The scatterplot of unigene and miRNA expression shows the positive correlation between transcriptome data and real-time qRT-PCR results. (PDF 110 kb)

Additional file 20: Table S18. Expression profiles of unigenes/miRNAs measured by RNA/small RNA sequencing and real-time qRT-PCR. (XLSX 54 kb)

Additional file 21: Figure S6. Real-time qRT-PCR validation of putative target genes at different germination stages. BlDS represents black dry seed, BlIS represents black imbibed seed, BlS represents seedlings germinated from black seed, BrDS represents brown dry seed, BrIS represents brown imbibed seed, BrS represents seedlings germinated from brown seed. (PDF 208 kb)

Additional file 22: Table S19. Unigenes involved in photosynthesis-antenna proteins pathway identified by KEGG enrichment. (XLSX 12 kb)

Additional file 23: Table S20. Unigenes involved in photosynthesis pathway identified by KEGG enrichment. (XLSX 56 kb)

Additional file 24: Table S21. The primers used in this study. (XLSX 10 kb)

## Abbreviations

ABA: Abscisic acid
Bl: Black
Br: Brown
CDS: Coding region sequences
COG: Clusters of orthologous groups
DE: Differentially expressed
DEG: Differentially expressed genes
DS: Dry seed
FC: Fold change
GA: Gibberellic acid
GO: Gene ontology
IS: Imbibed seed
KEGG: Kyoto encyclopedia of genes and genome
MFE: Minimal folding free energy
mfe_ratio: miRNA-transcript duplex free energy ratio
MFEI: Minimal folding free energy indice
Nr: Non-redundant
qRT-PCR: Real-time quantitative reverse transcription-PCR
RACE: Rapid amplification of cDNA ends
RNA-seq: RNA sequence
RPKM: The reads per kilobase of transcript per million mapped reads
S: Seedling
sRNA-seq: Small RNA sequence
TAIR: Arabidopsis information resource
TPM: The mapped reads were normalized to tags per million
