# Supplementary figures and images for: Transcriptome assembly in Suaeda aralocaspica to reveal the distinct temporal gene/miRNA alterations between the dimorphic seeds during germination
Source: BMC Genomics. 2017 Oct 19;18:806. doi: 10.1186/s12864-017-4209-1 (PMC5649071; doi:10.1186/s12864-017-4209-1)

A

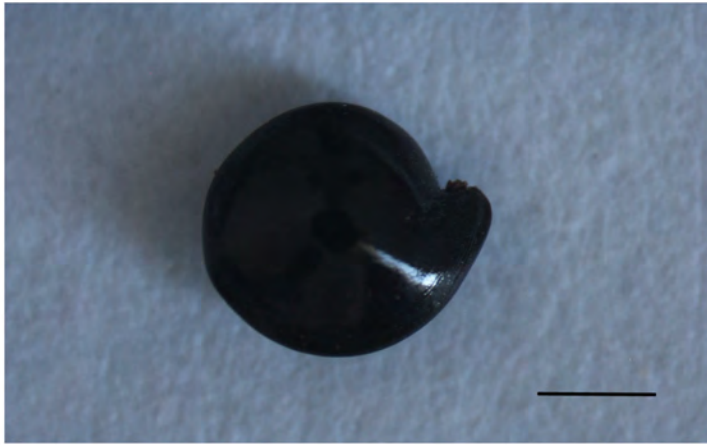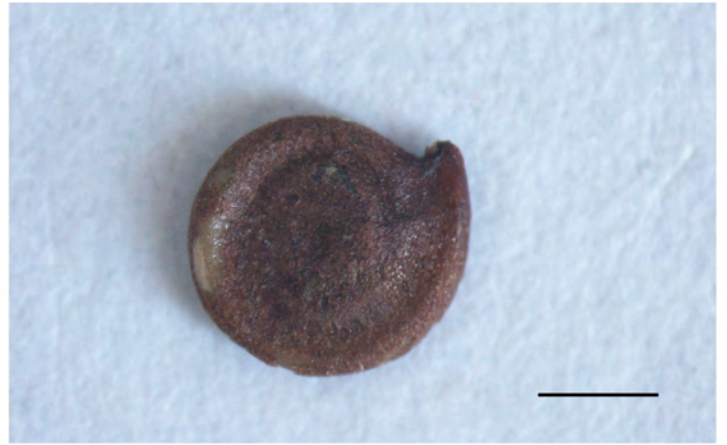

B

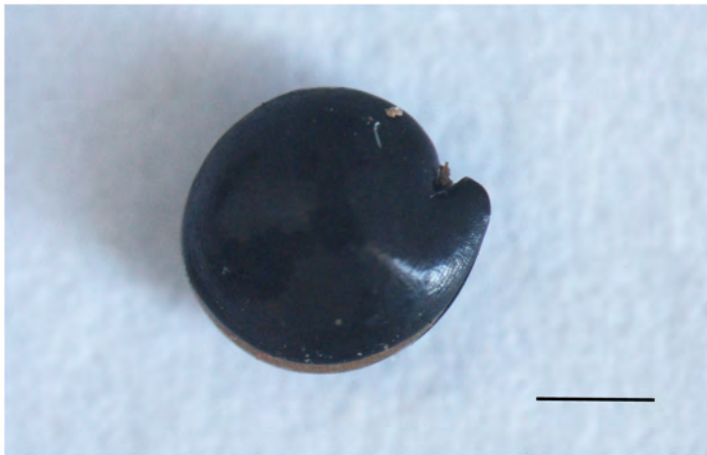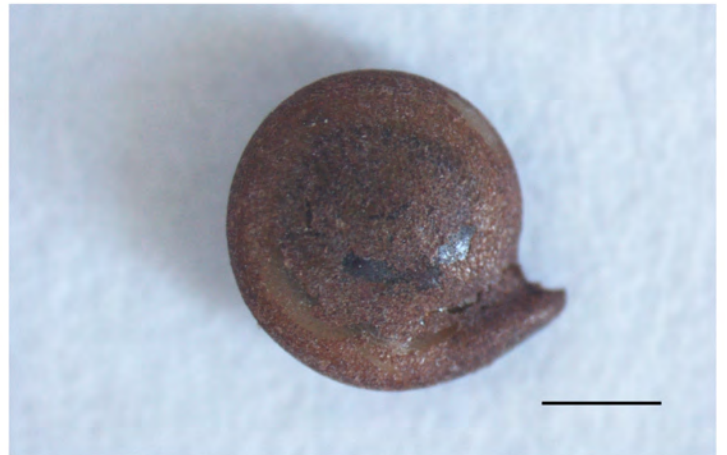

C

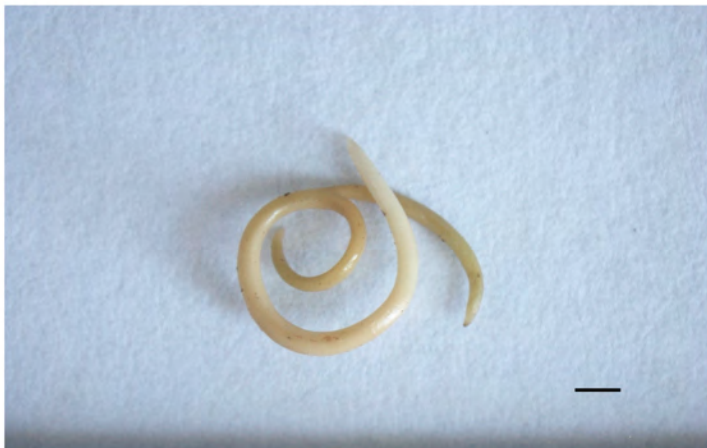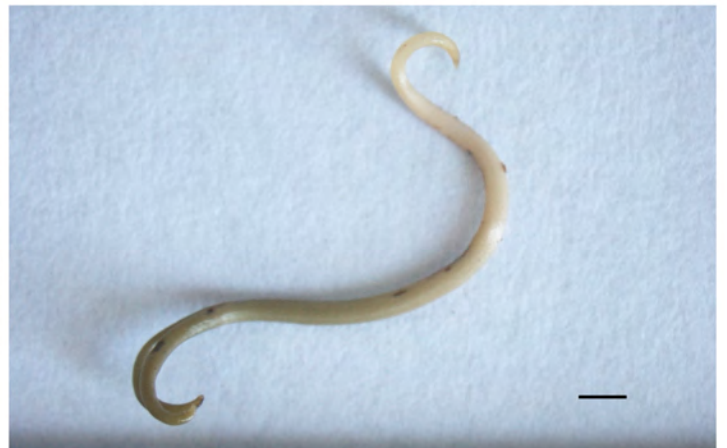

Black

Brown

Supplement: Supplementary file 1 — Morphology of seed germination in Suaeda aralocaspica. Bar = 1 mm. (PDF 13983 kb) [file 12864_2017_4209_MOESM1_ESM.pdf]

A

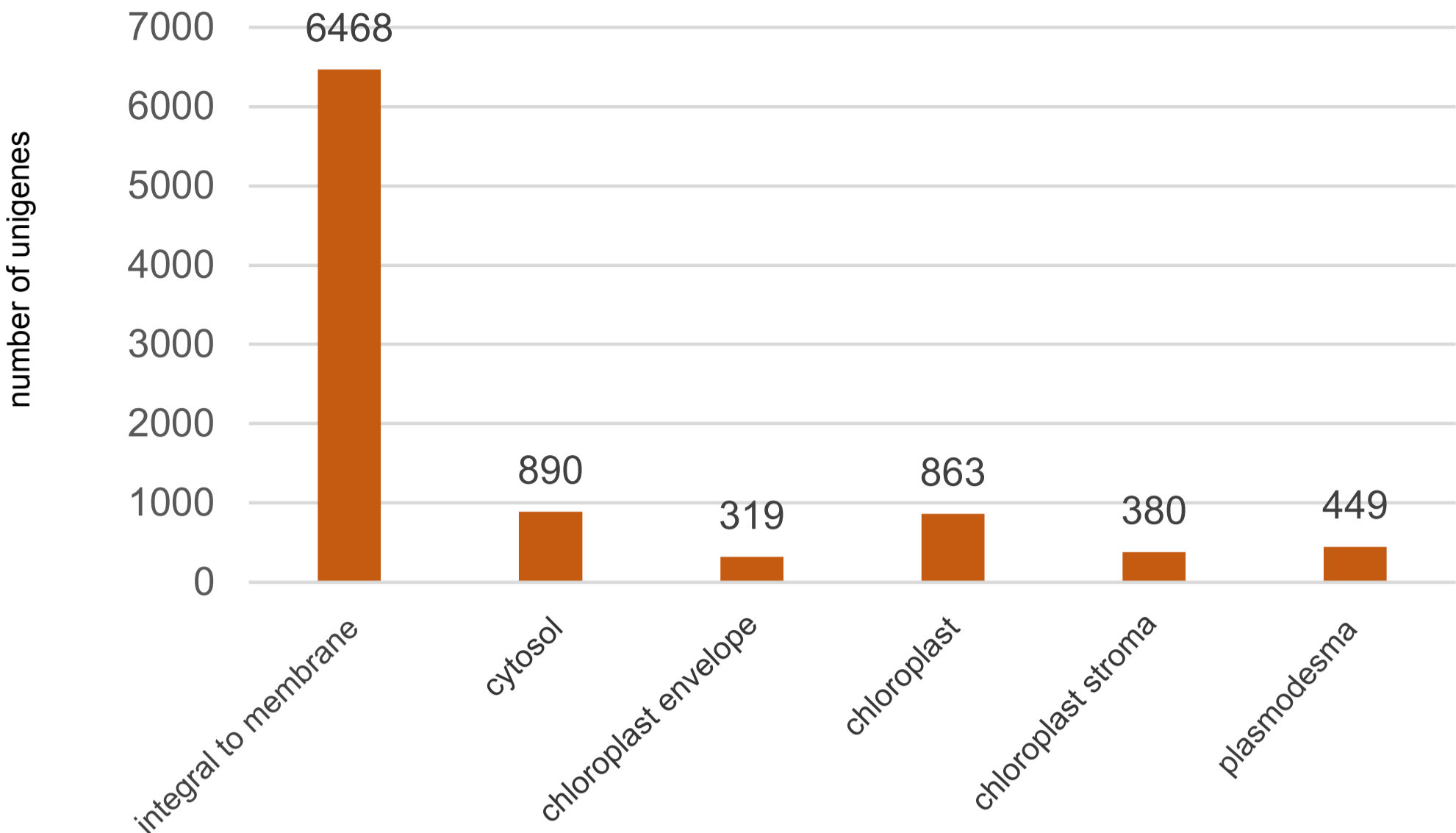

B

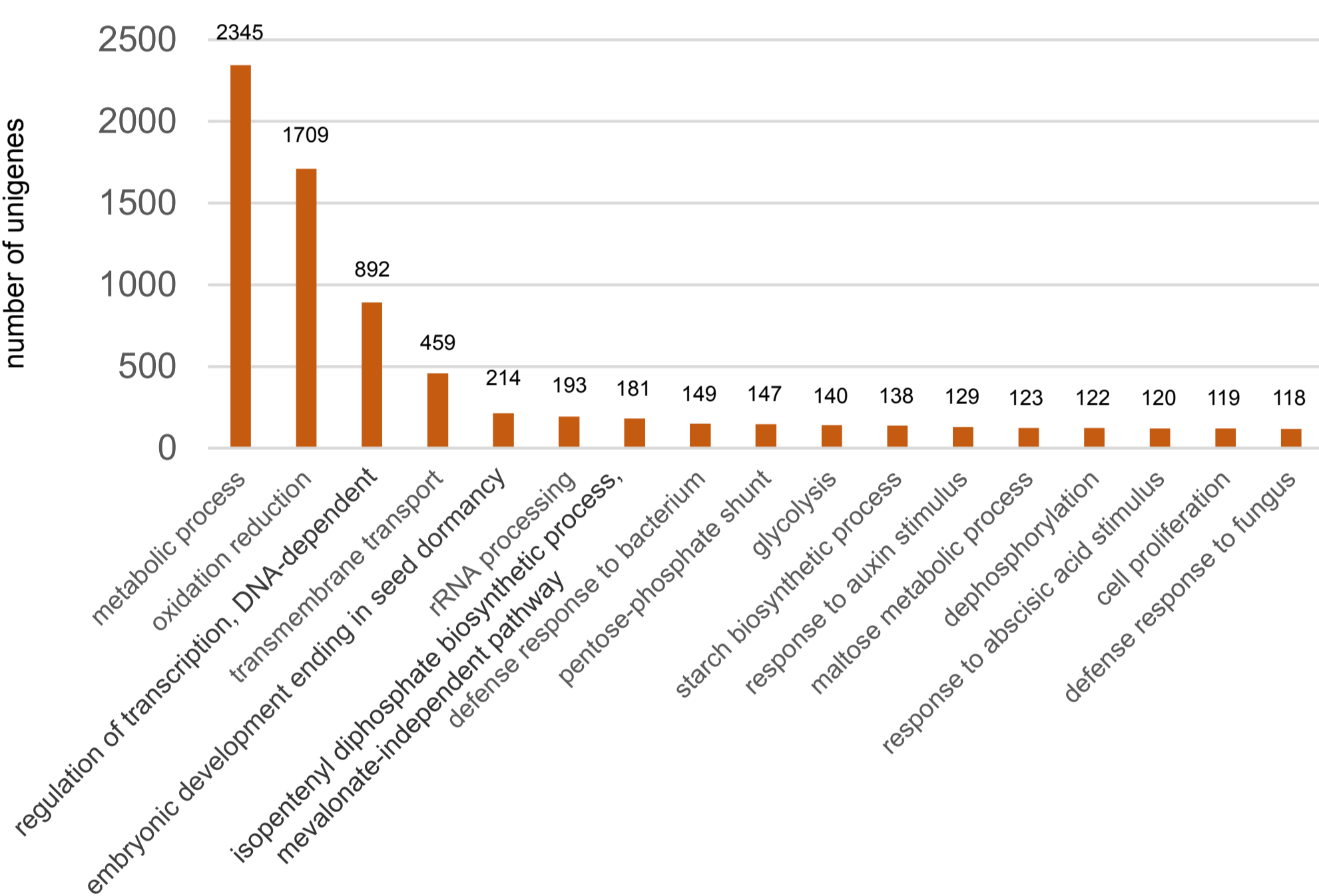

C

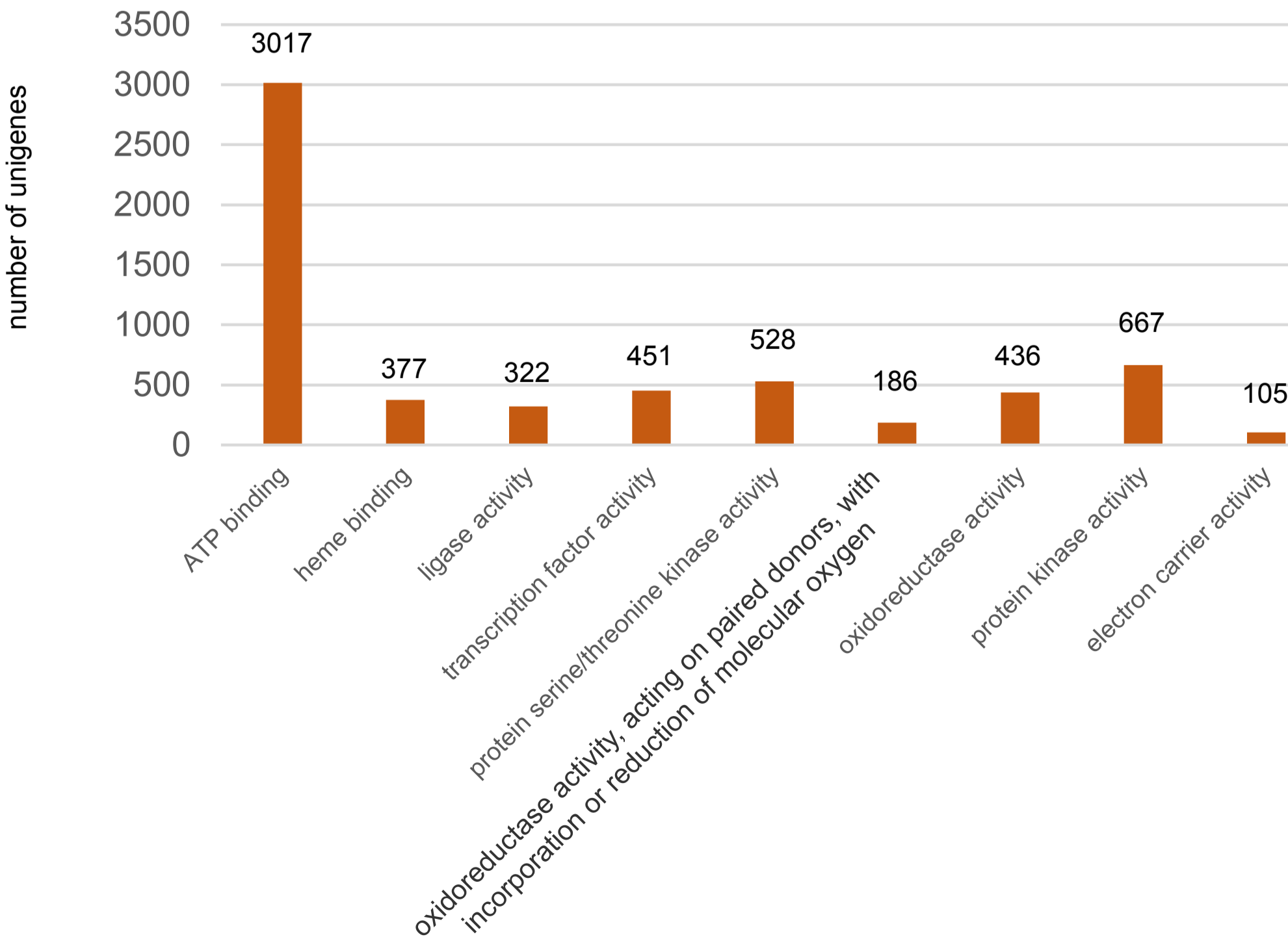

Supplement: Supplementary file 3 — GO functional classification of Suaeda aralocaspica unigenes within the category of cellular component (A), biological process (B) and molecular function (C). The number of unigenes enriched in each subcategory is indicated above each bar. (PDF 406 kb) [file 12864_2017_4209_MOESM3_ESM.pdf]

Transcript

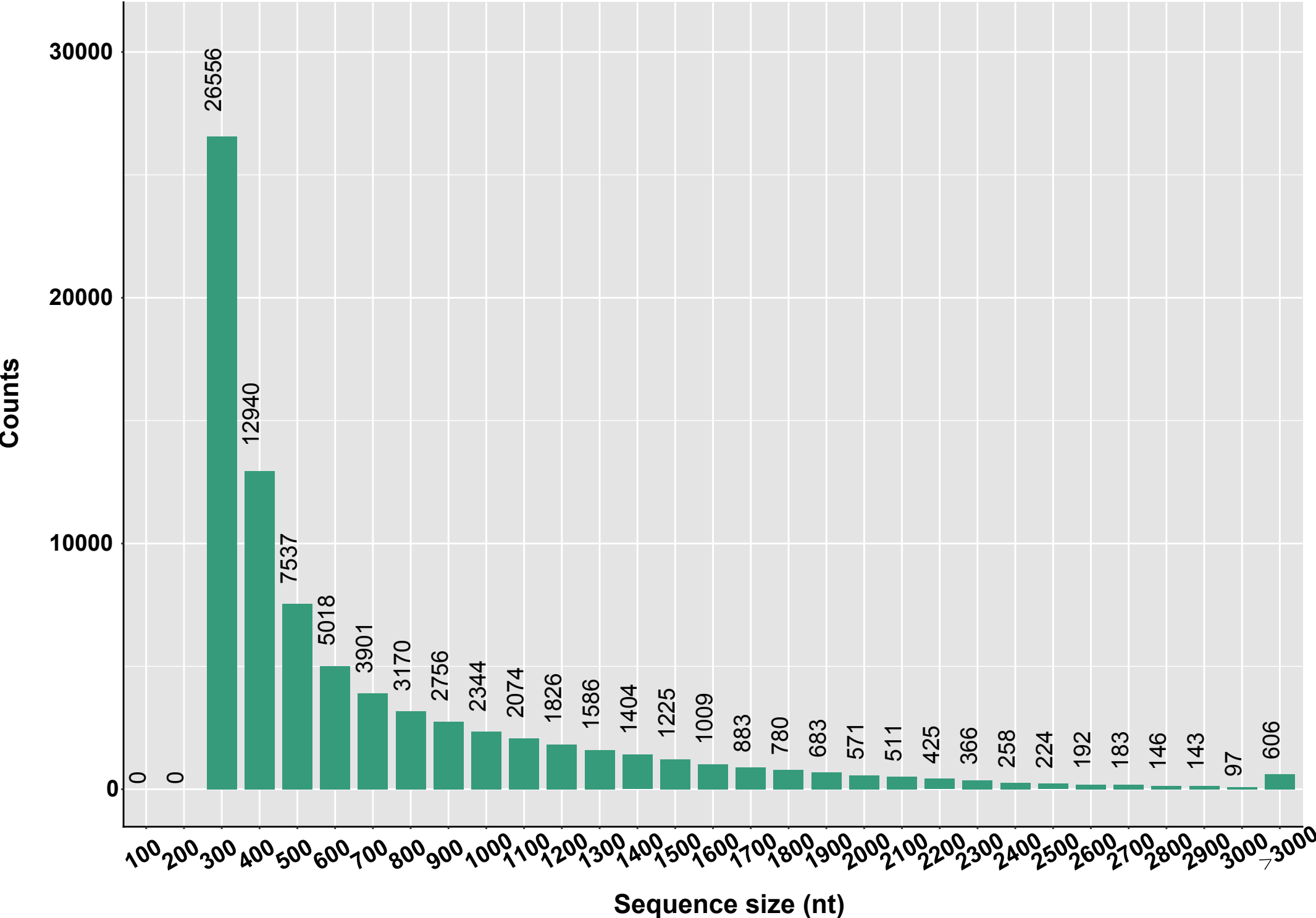

CDS

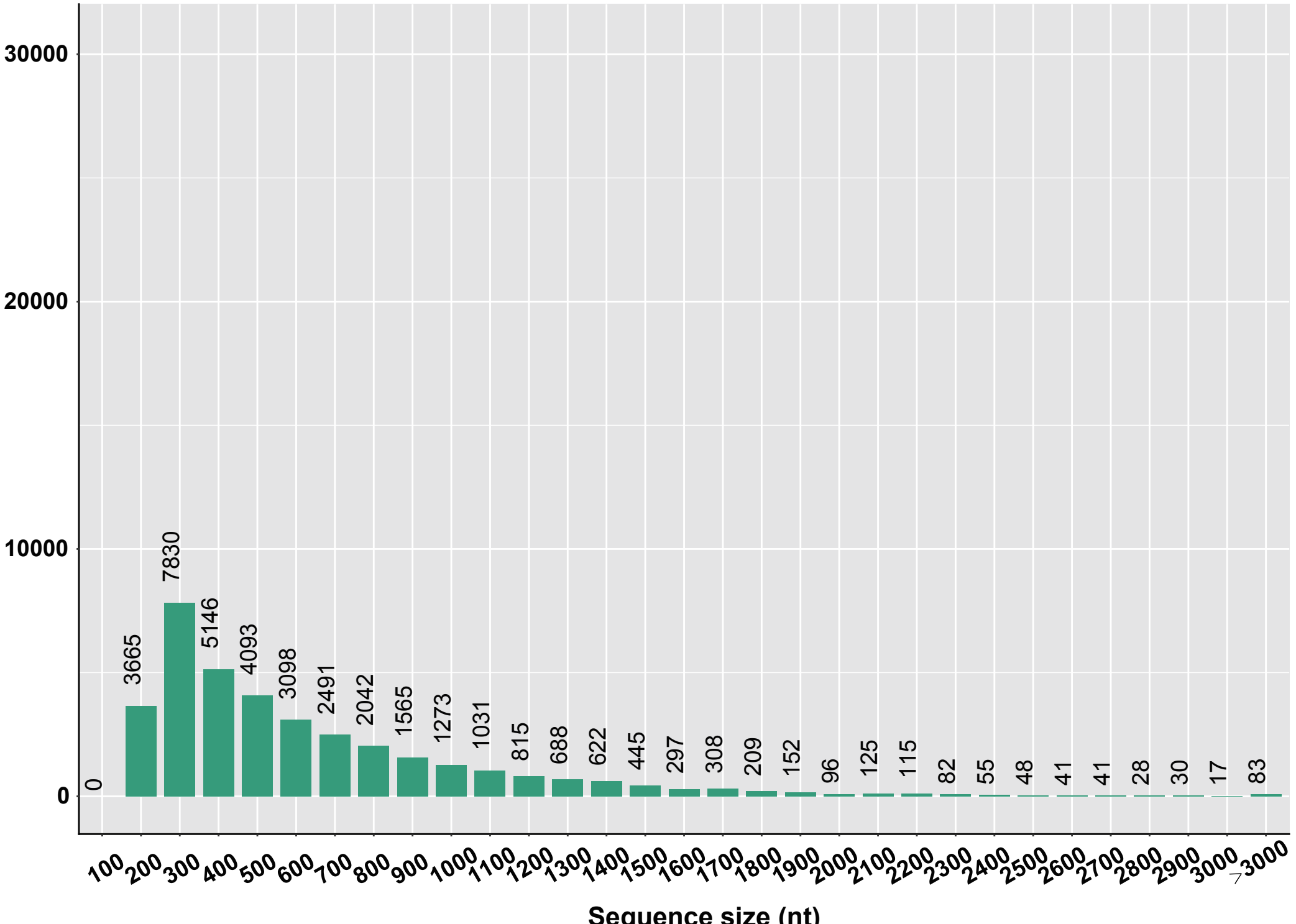

Supplement: Supplementary file 4 — Length distribution of Suaeda aralocaspica assembled transcripts and coding region sequences (CDS) of the transcripts. nt, nucleotides. (PDF 507 kb) [file 12864_2017_4209_MOESM4_ESM.pdf]

A

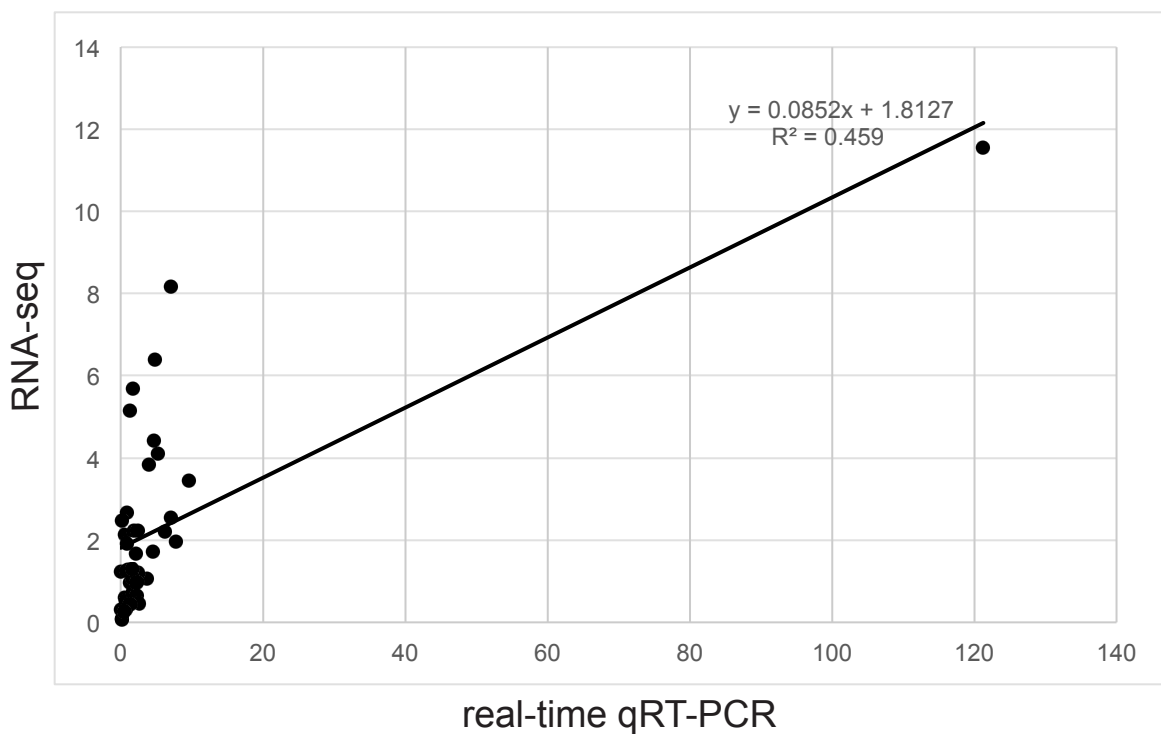

B

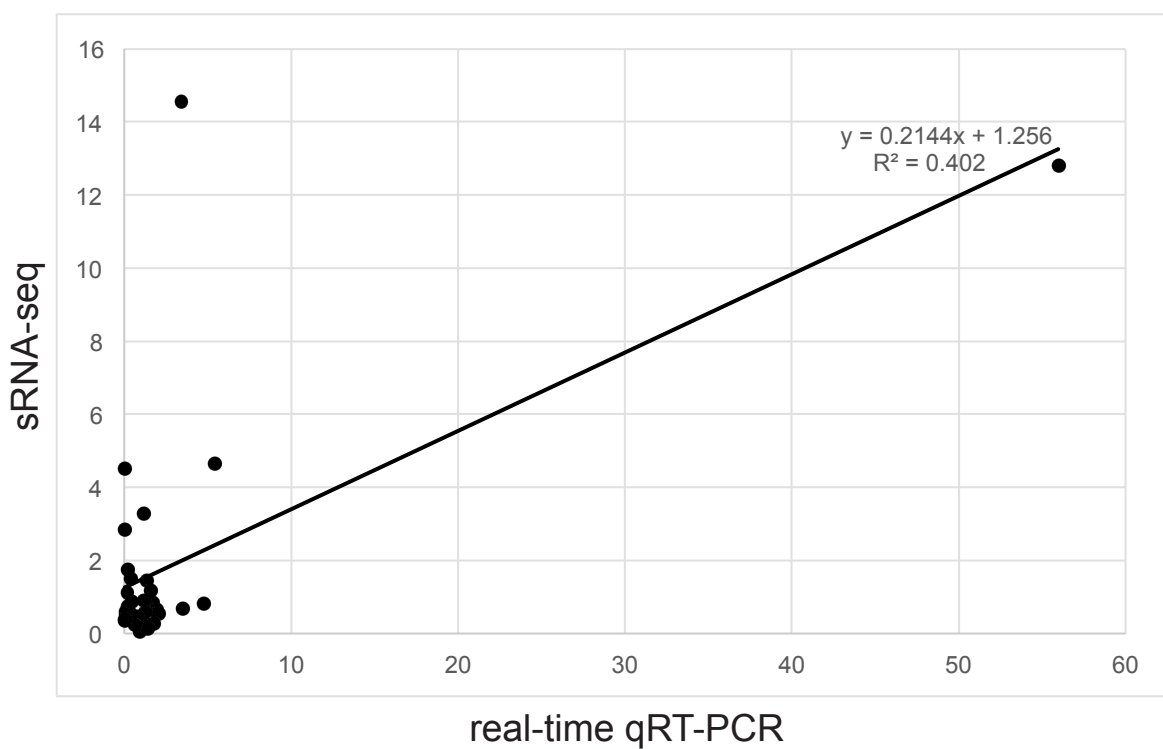

Supplement: Supplementary file 19 — Validation of the expression profiles of selected unigenes (A) and miRNAs (B). The scatterplot of unigene and miRNA expression shows the positive correlation between transcriptome data and real-time qRT-PCR results. (PDF 110 kb) [file 12864_2017_4209_MOESM19_ESM.pdf]

Additional file 21: Figure S6

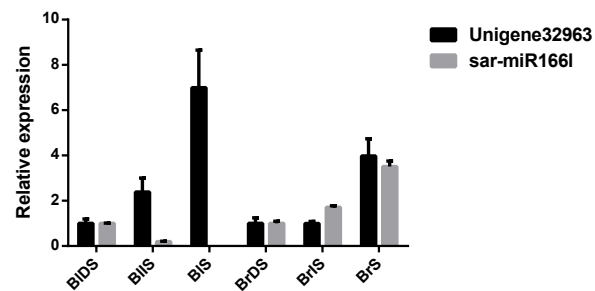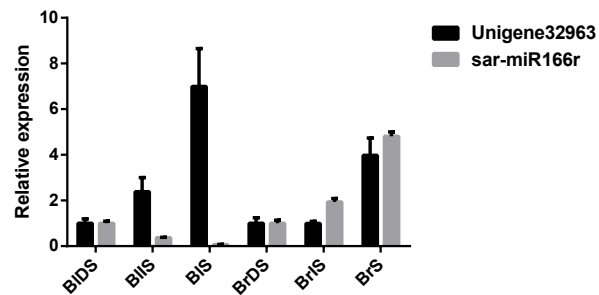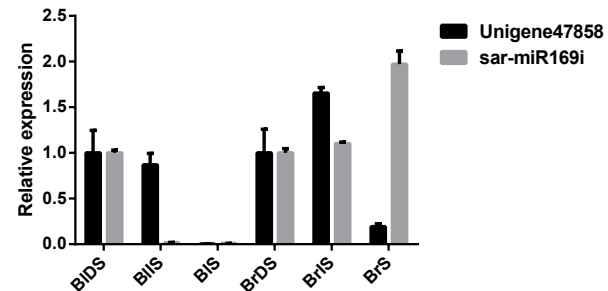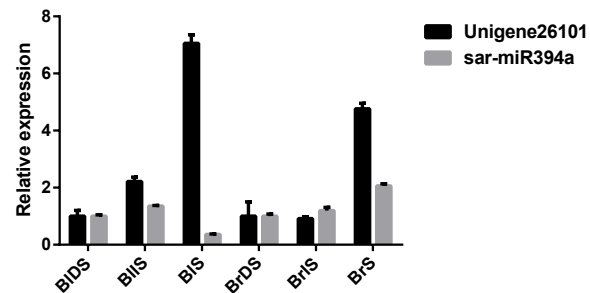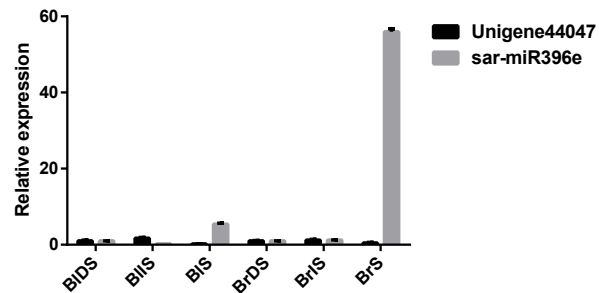

Supplement: Supplementary file 21 — Real-time qRT-PCR validation of putative target genes at different germination stages. BlDS represents black dry seed, BlIS represents black imbibed seed, BlS represents seedlings germinated from black seed, BrDS represents brown dry seed, BrIS represents brown imbibed seed, BrS represents seedlings germinated from brown seed. (PDF 208 kb) [file 12864_2017_4209_MOESM21_ESM.pdf]
