# Supplementary material for: Transcriptome assembly in Suaeda aralocaspica to reveal the distinct temporal gene/miRNA alterations between the dimorphic seeds during germination
Source: BMC Genomics. 2017 Oct 19;18:806. doi: 10.1186/s12864-017-4209-1 (PMC5649071; doi:10.1186/s12864-017-4209-1)
Supplement: Supplementary file 2 — Statistics of RNA sequencing reads. Table S2. Summary of de novo sequence assembly using Trinity. Table S3. Annotation on assembled transcripts by different public databases. Table S11. Statistics of small RNA sequences from the six small RNA libraries. (DOCX 59 kb) [file 12864_2017_4209_MOESM2_ESM.docx]

Table S1 Statistics of RNA sequencing reads.

| Reads preparation | Libraries | Number of reads | Total reads |
| --- | --- | --- | --- |
| Raw reads | BlDS | 35,891,486 | 225,861,504 |
|  | BlIS | 37,113,558 |  |
|  | BlS | 39,939,590 |  |
|  | BrDS | 40,245,214 |  |
|  | BrIS | 37,000,556 |  |
|  | BrS | 35,671,100 |  |
| FASTX-Toolkit process | BlDS | 31,756,558 | 201,609,259 |
|  | BlIS | 36,342,924 |  |
|  | BlS | 31,513,784 |  |
|  | BrDS | 36,103,834 |  |
|  | BrIS | 32,120,631 |  |
|  | BrS | 33,771,528 |  |

BlDS represents black dry seed, BlIS represents black imbibed seed, BlS represents seedlings germinated from black seed, BrDS represents brown dry seed, BrIS represents brown imbibed seed, BrS represents seedlings germinated from brown seed.

Table S2 Summary of *de novo* sequence assembly using Trinity.

|  | Total nucleotides (nt) | All numbers | Average length (bp) | N50 (bp) |
| --- | --- | --- | --- | --- |
| Total clean reads | 17,011,844,081 | 201,609,259 | 84 |  |
| Total unigenes | 69,704,197 | 106,171 | 657 | 980 |
| Non-redundant unigenes | 51,415,356 | 79,414 | 647 | 963 |
| Q20 percentage | 97.43% |  |  |  |

Table S3 Annotation on assembled transcripts by different public databases.

| Database | Number | Annotation rate |
| --- | --- | --- |
| Public database | 44,327 | 55.82% |
| Nr | 43,303 | 54.53% |
| KEGG | 38,754 | 48.80% |
| Swiss-Prot | 27,399 | 34.50% |
| COG | 11,162 | 14.06% |
| *Beta vulgaris* | 44,041 | 55.46% |
| BLASTX^1^ | 41,374 | 52.10% |
| BLAT^2^ | 10,853 | 13.67% |
| *Populus euphratica* | 36,050 | 45.40% |
| BLASTX^1^ | 35,237 | 44.37% |
| BLAT^2^ | 3,679 | 4.63% |
| Total | 45,796 | 57.67% |

^1^means the transcripts annotated by whole protein sequences using BLASTX.

^2^means the transcripts annotated by whole genome using BLAT.

Table S11 Statistics of small RNA sequences from the six small RNA libraries.

| Category | BlDS | BlIS | BlS | BrDS | BrIS | BrS |
| --- | --- | --- | --- | --- | --- | --- |
| Raw reads | 6,034,868 | 6,121,557 | 6,882,624 | 5,469,210 | 6,222,416 | 5,727,307 |
| Clean reads (18-30 nt) | 3,787,572 | 2,979,788 | 3,567,931 | 3,220,810 | 2,483,312 | 2,496,365 |
| Unique sequence reads (18-30 nt) | 1,603,623(42.34%)* | 1,220,278(40.95%) | 1,352,423(37.90%) | 1,155,047(35.86%) | 1,166,618(46.98%) | 899,193(36.02%) |
| Match to *Suaeda aralocaspica* mRNA transcriptome | 2,026,696(53.53%) | 1,517,595(50.93%) | 1,864,179(52.25%) | 1,386,419(43.05%) | 1,398,553(56.32%) | 1,127,307(45.16%) |
| Match to known miRNAs | 397,063 | 223,997 | 518,491 | 149,378 | 174,448 | 431,897 |
| Match to known *Salicornia europaea* sRNAs | 23 | 2 | 14 | 2 | 3 | 19 |

BlDS represents black dry seed, BlIS represents black imbibed seed, BlS represents seedlings germinated from black seed, BrDS represents brown dry seed, BrIS represents brown imbibed seed, BrS represents seedlings germinated from brown seed.

*Read counts in the middle section were expressed in numbers (left) or as a percentage of the clean reads (18-30 nt) (right).
