# Supplementary material for: Transcriptome assembly in Suaeda aralocaspica to reveal the distinct temporal gene/miRNA alterations between the dimorphic seeds during germination
Source: BMC Genomics. 2017 Oct 19;18:806. doi: 10.1186/s12864-017-4209-1 (PMC5649071; doi:10.1186/s12864-017-4209-1)
Supplement: Supplementary file 15 — The secondary structures of Suaeda aralocaspica miRNAs predicted by Mfold. The mature sequences of miRNA were highlighted in yellow. (PDF 2399 kb) [file 12864_2017_4209_MOESM15_ESM.pdf]

Additional file 15: Figure S4

Output of sir_graph (©)
mfold_util 4.7

Created Mon Aug 29 22:33:53 2016

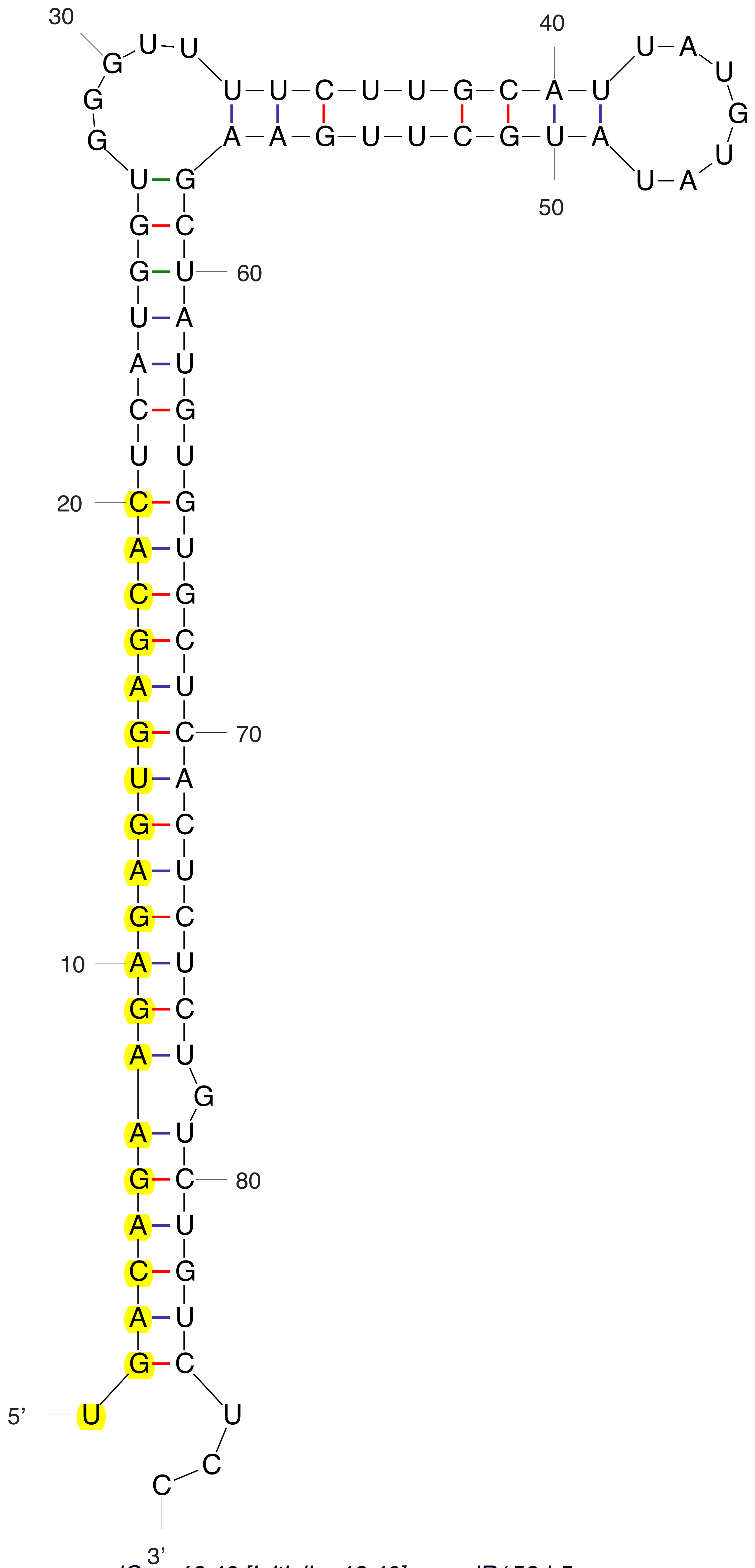

dG = -46.40 [Initially -46.40] sar-miR156d-5p

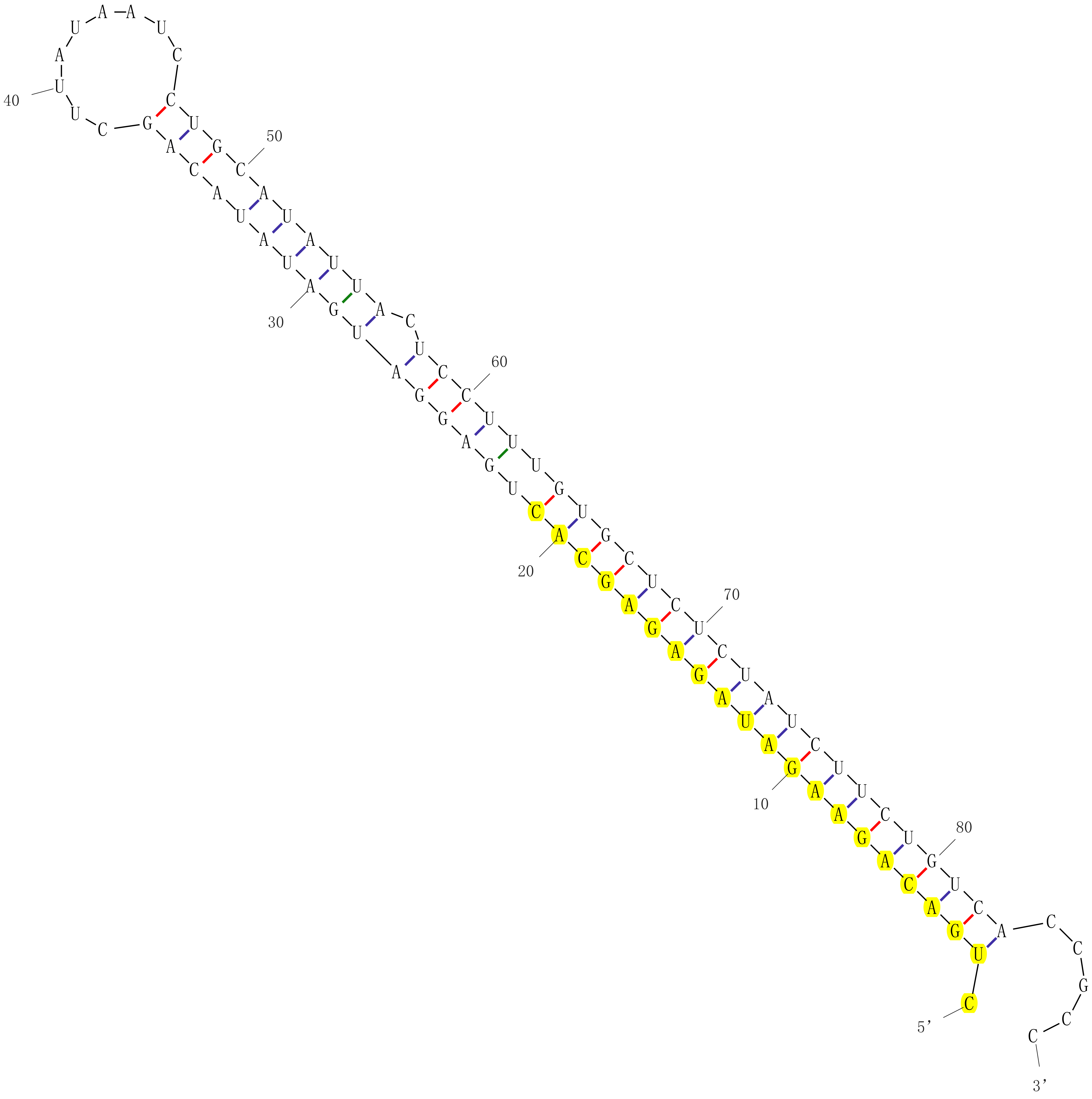

dG = -48.50 [Initially -48.50] sar-miR156t-5p

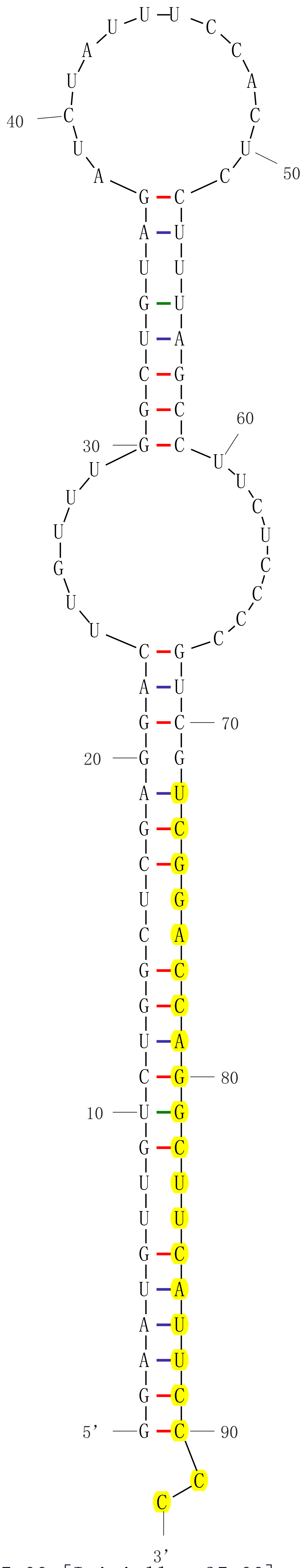

dG = -35.00 [Initially -35.00] sar-miR166b-3p

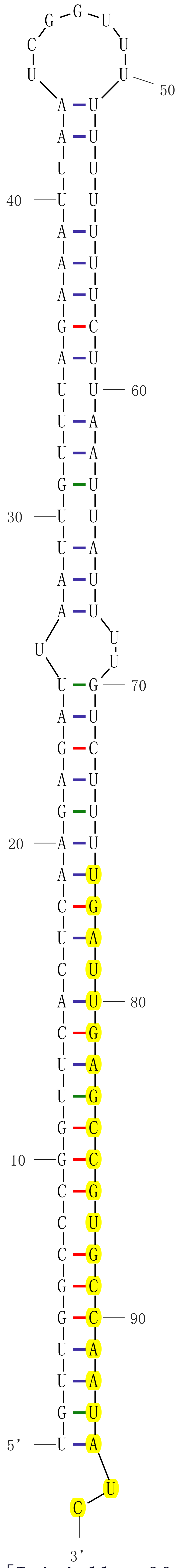

dG = -36.20 [Initially -36.20] sar-miR171e-3p

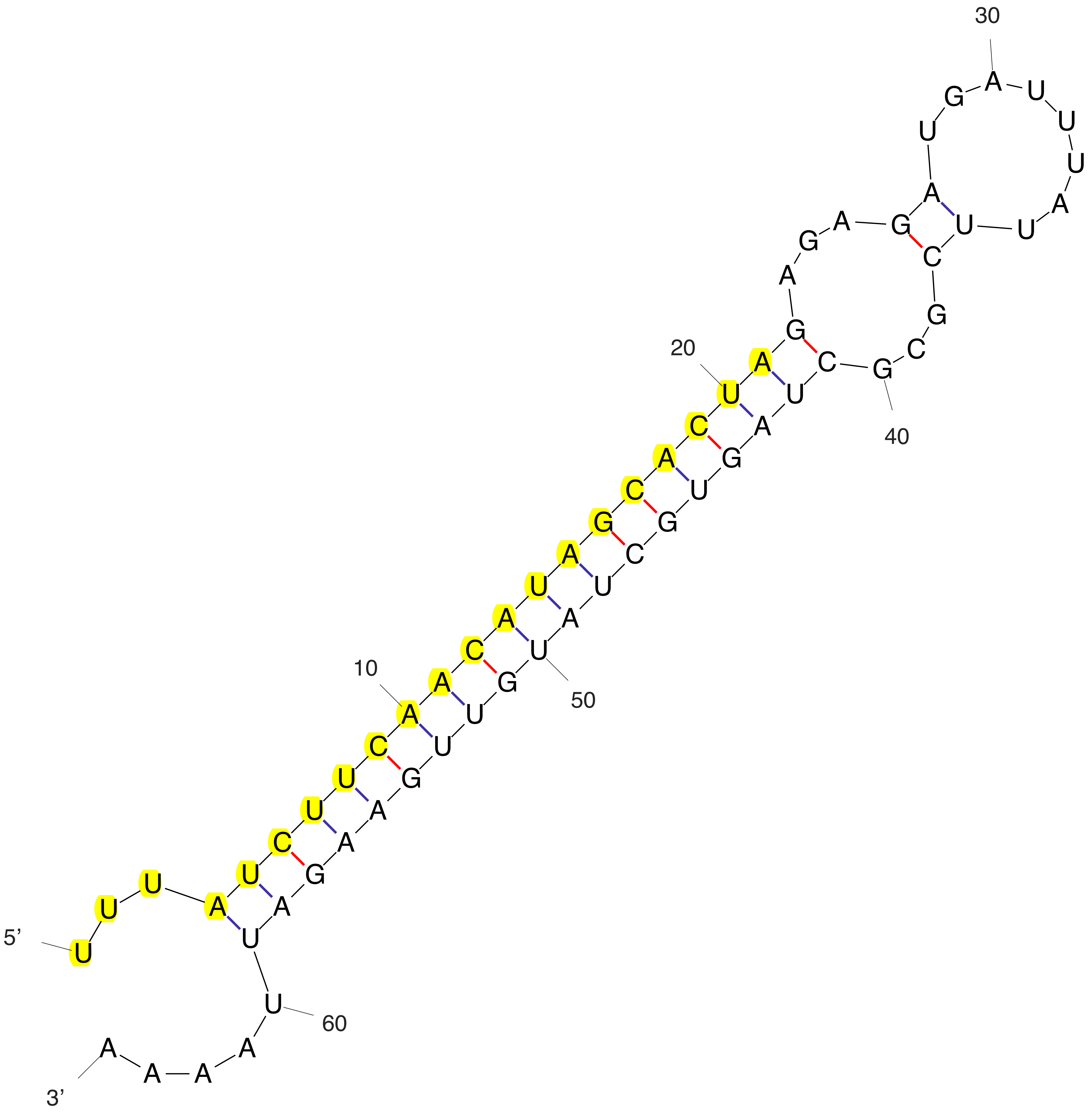

dG = -31.80 [Initially -31.80] sar-miR1a

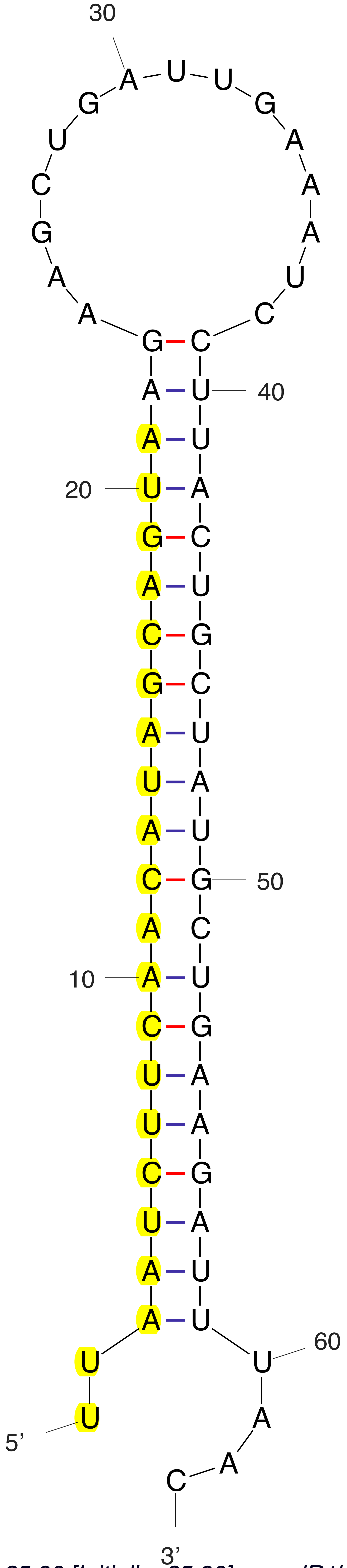

dG = -25.90 [Initially -25.90] sar-miR1b

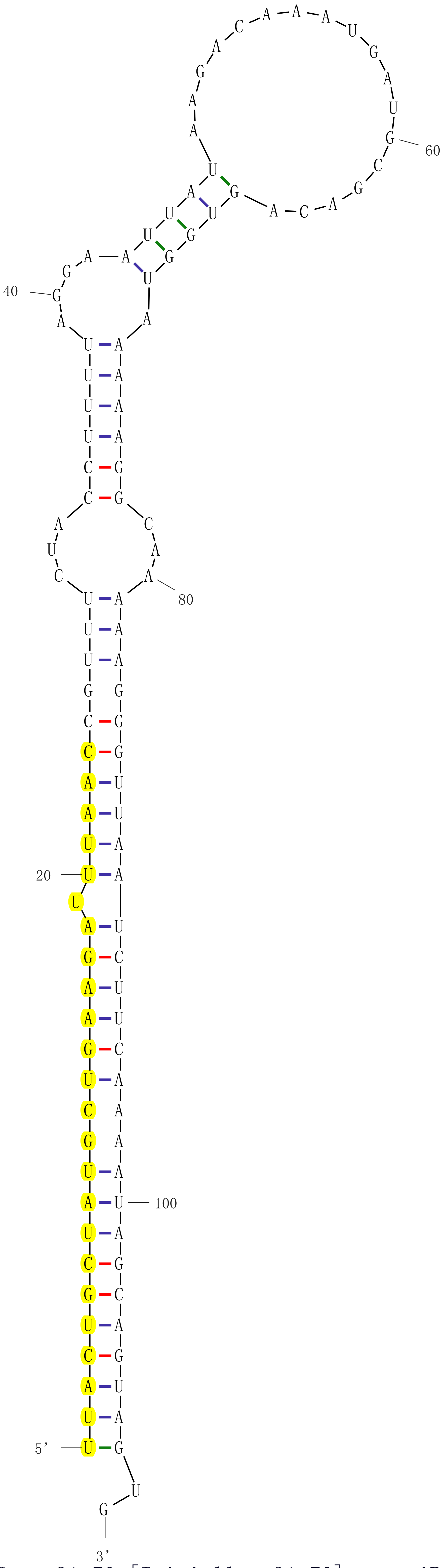

dG = -31.70 [Initially -31.70] sar-miR2

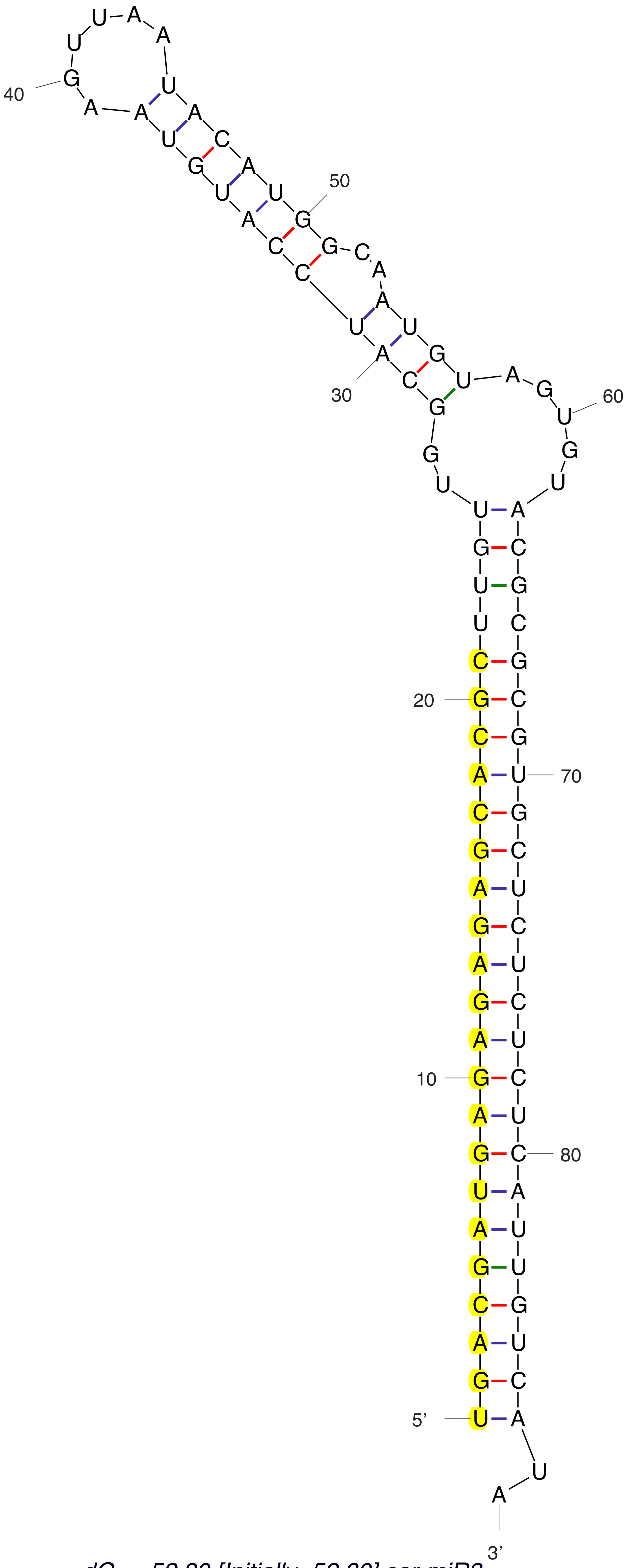

dG = -52.80 [Initially -52.80] sar-miR3

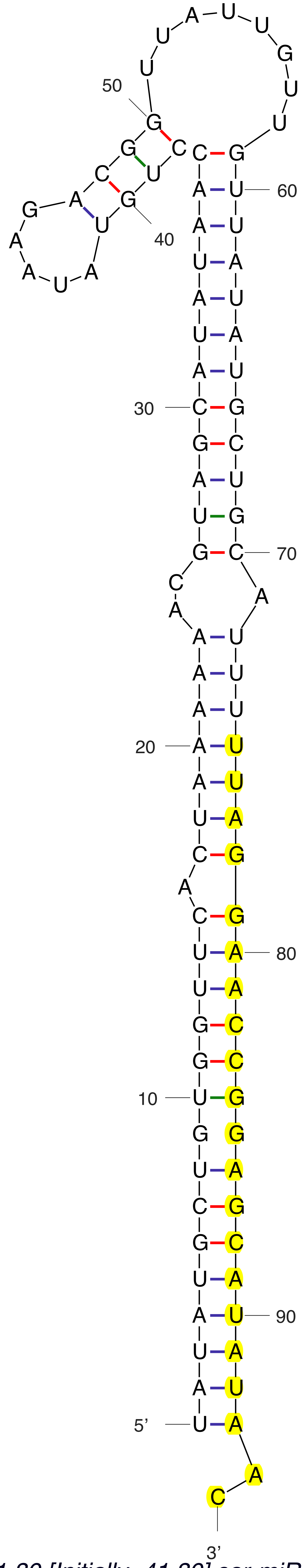

dG = -41.80 [Initially -41.80] sar-miR4

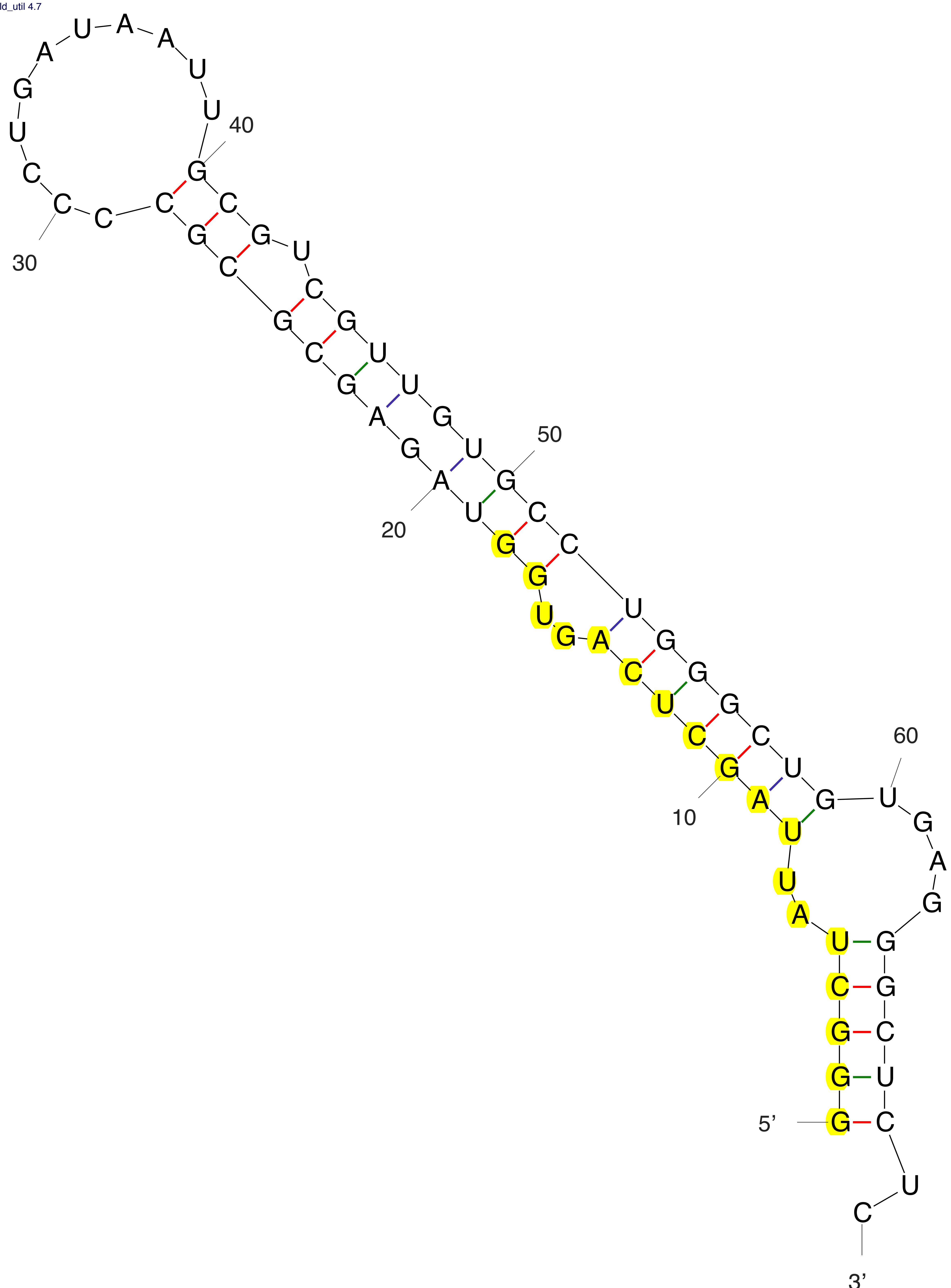

dG = -28.90 [Initially -28.90] sar-miR5

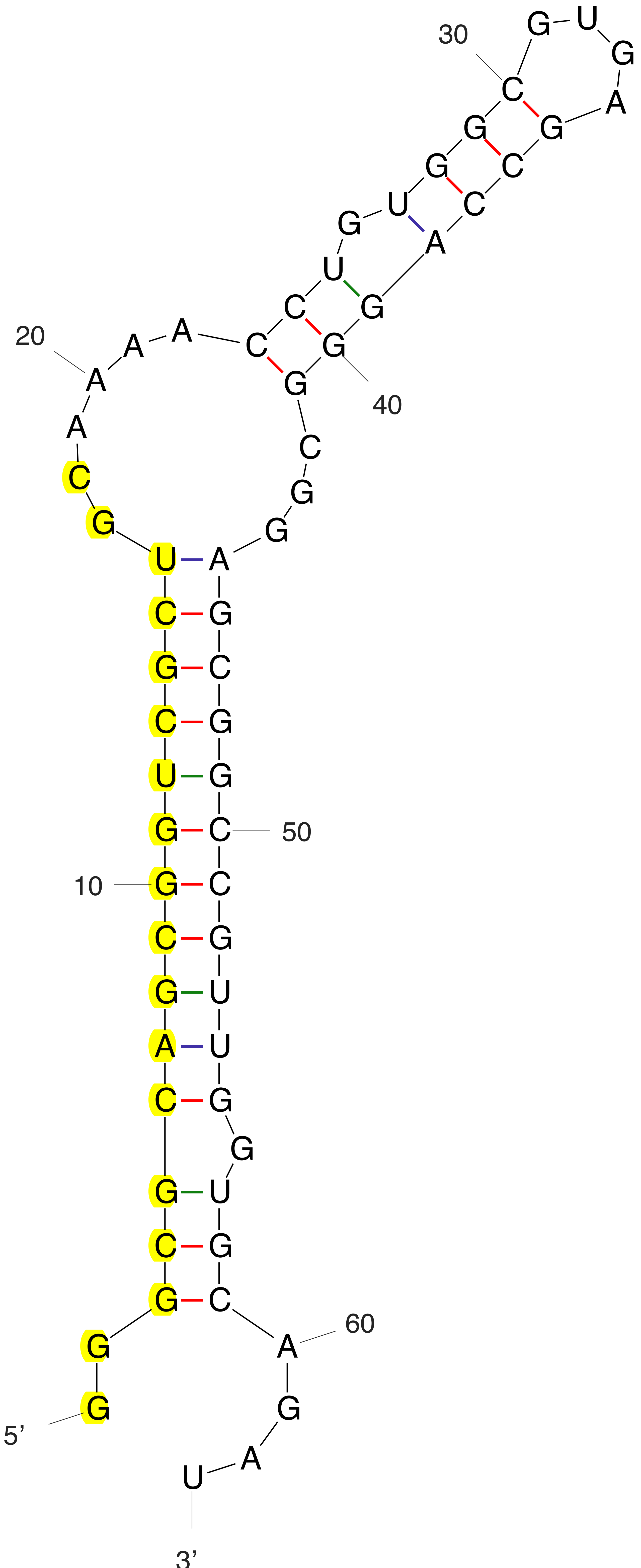

dG = -34.00 [Initially -34.00] sar-miR6a

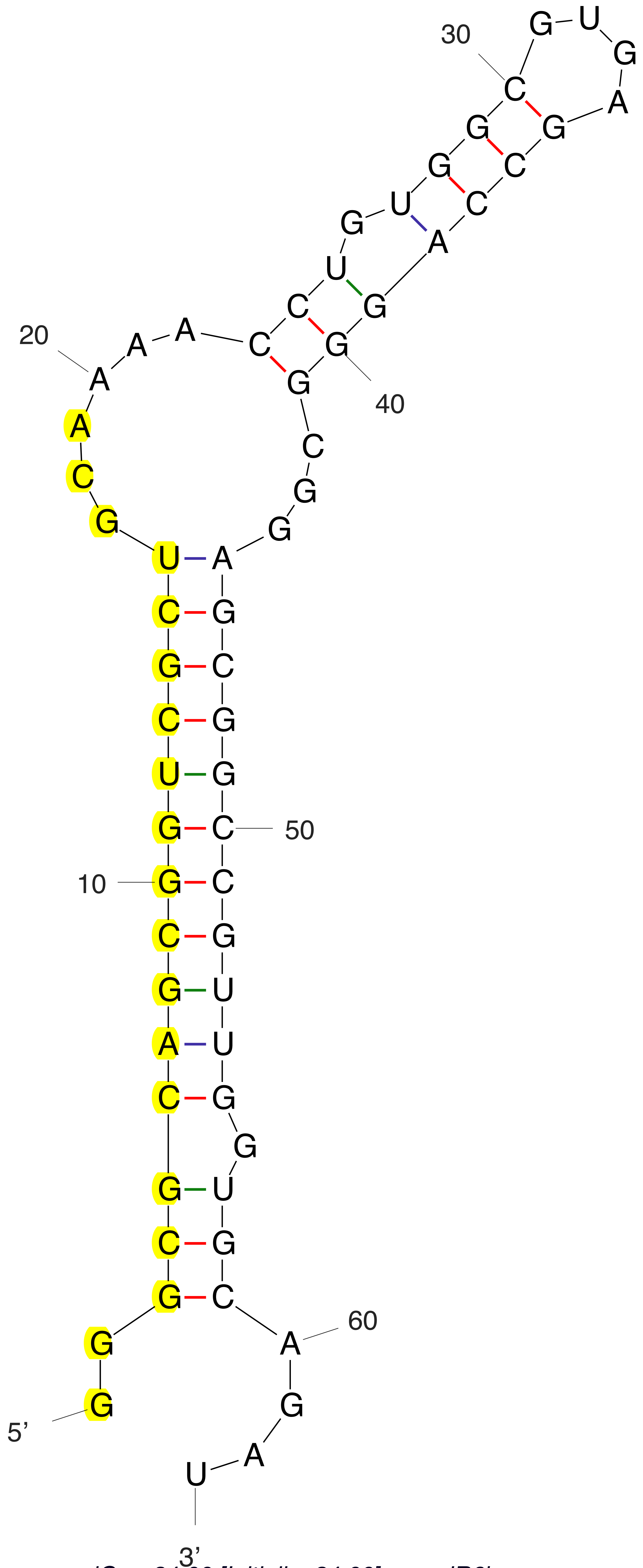

dG = -34.00 [Initially -34.00] sar-miR6b

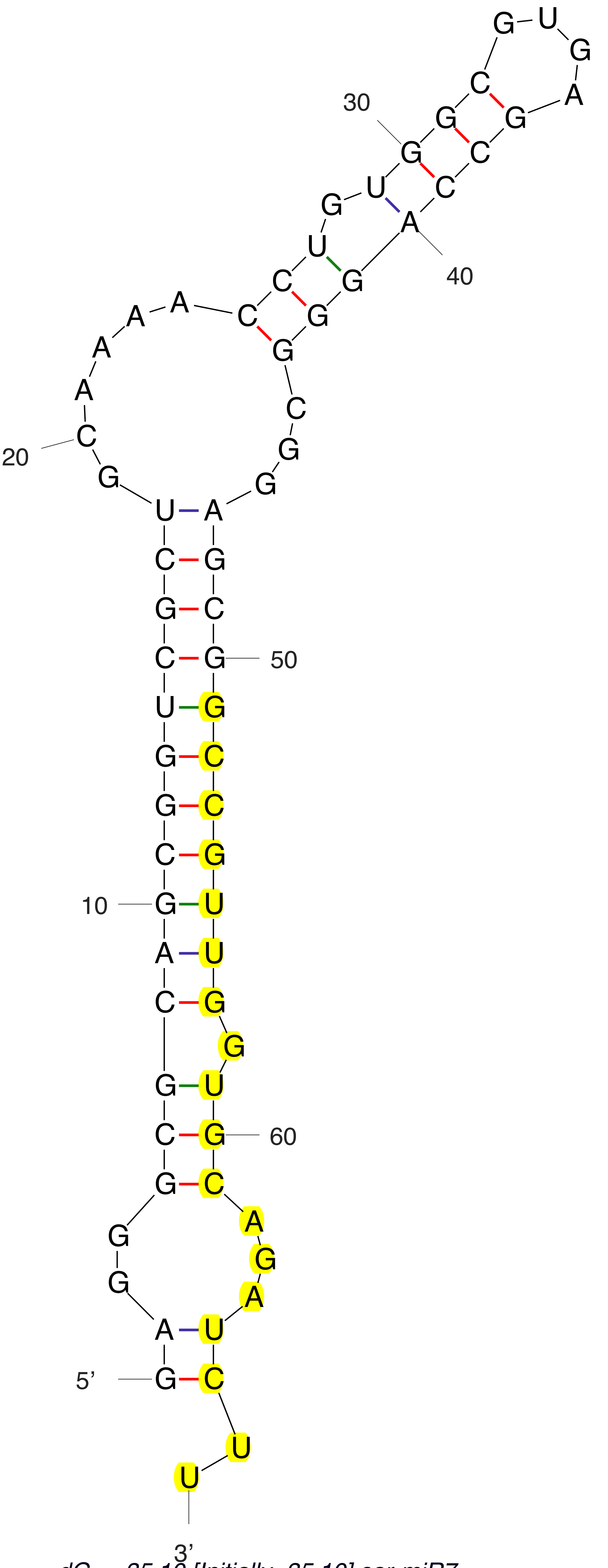

dG = -35.10 [Initially -35.10] sar-miR7

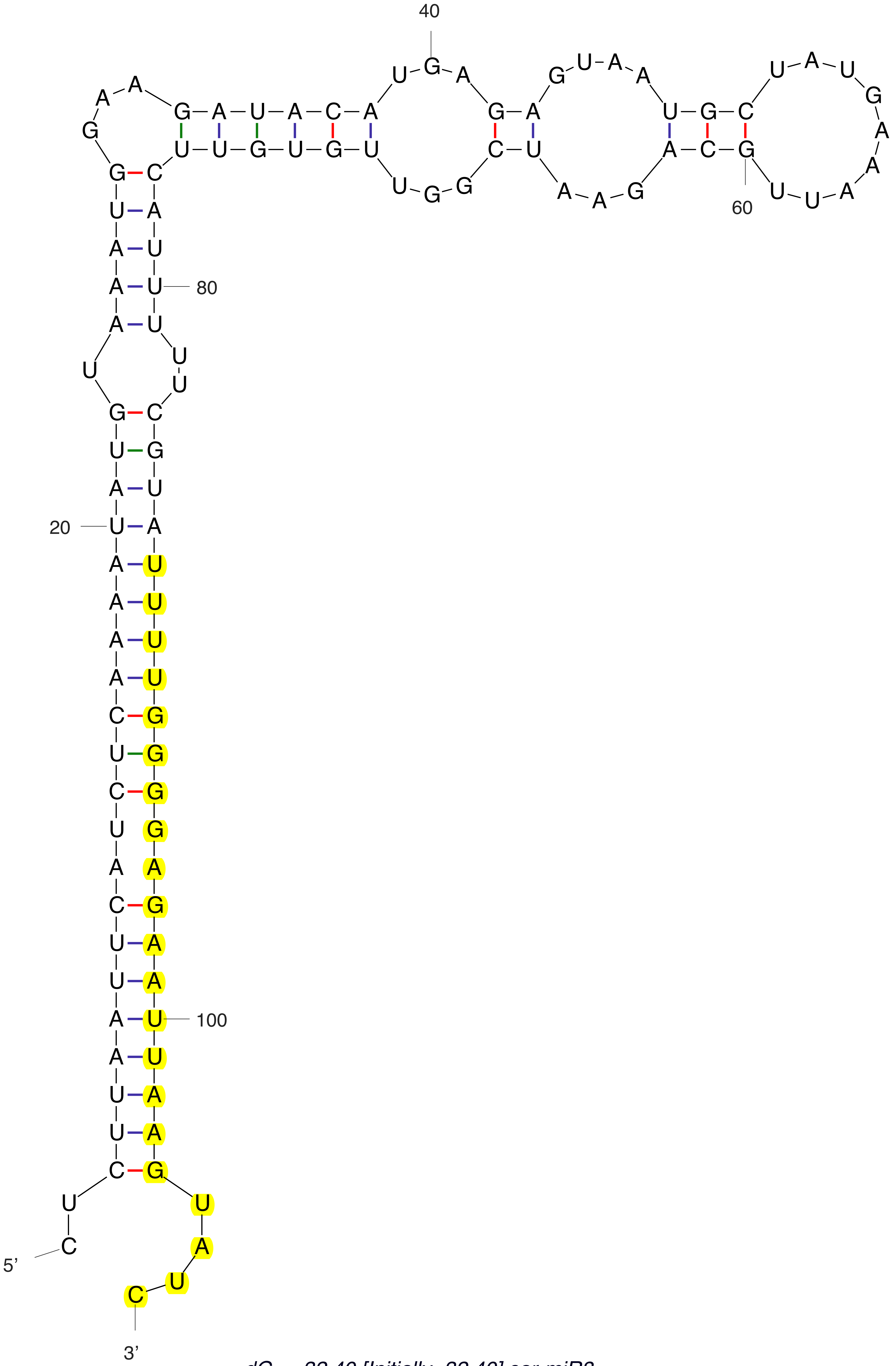

dG = -32.40 [Initially -32.40] sar-miR8

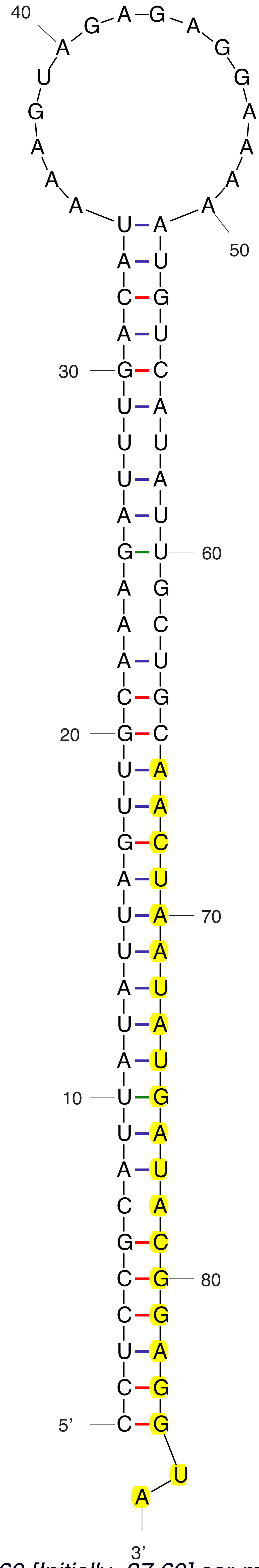

dG = -37.60 [Initially -37.60] sar-miR9

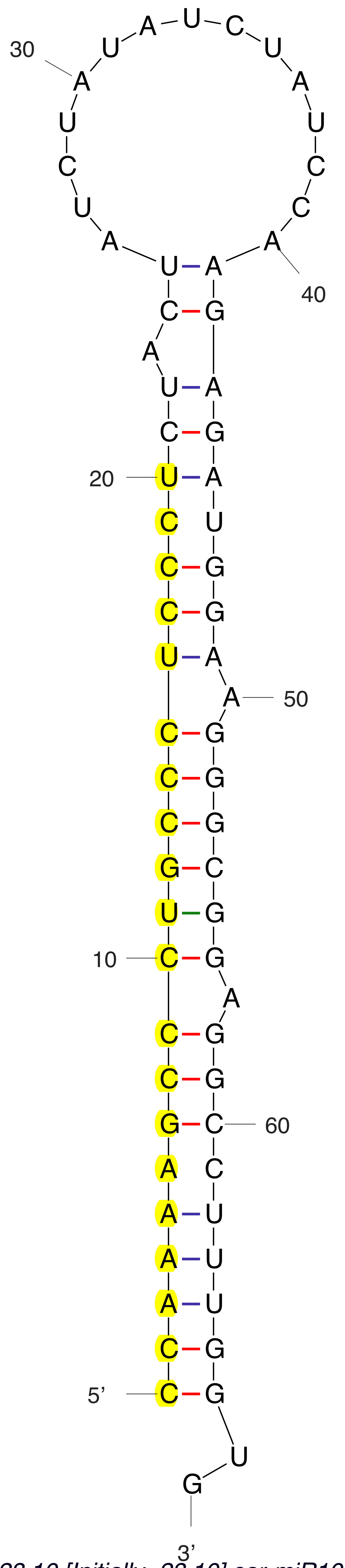

dG = -28.10 [Initially -28.10] sar-miR10

Output of sir_graph (©)
mfold_util 4.7

Created Tue Aug 23 21:58:52 2016

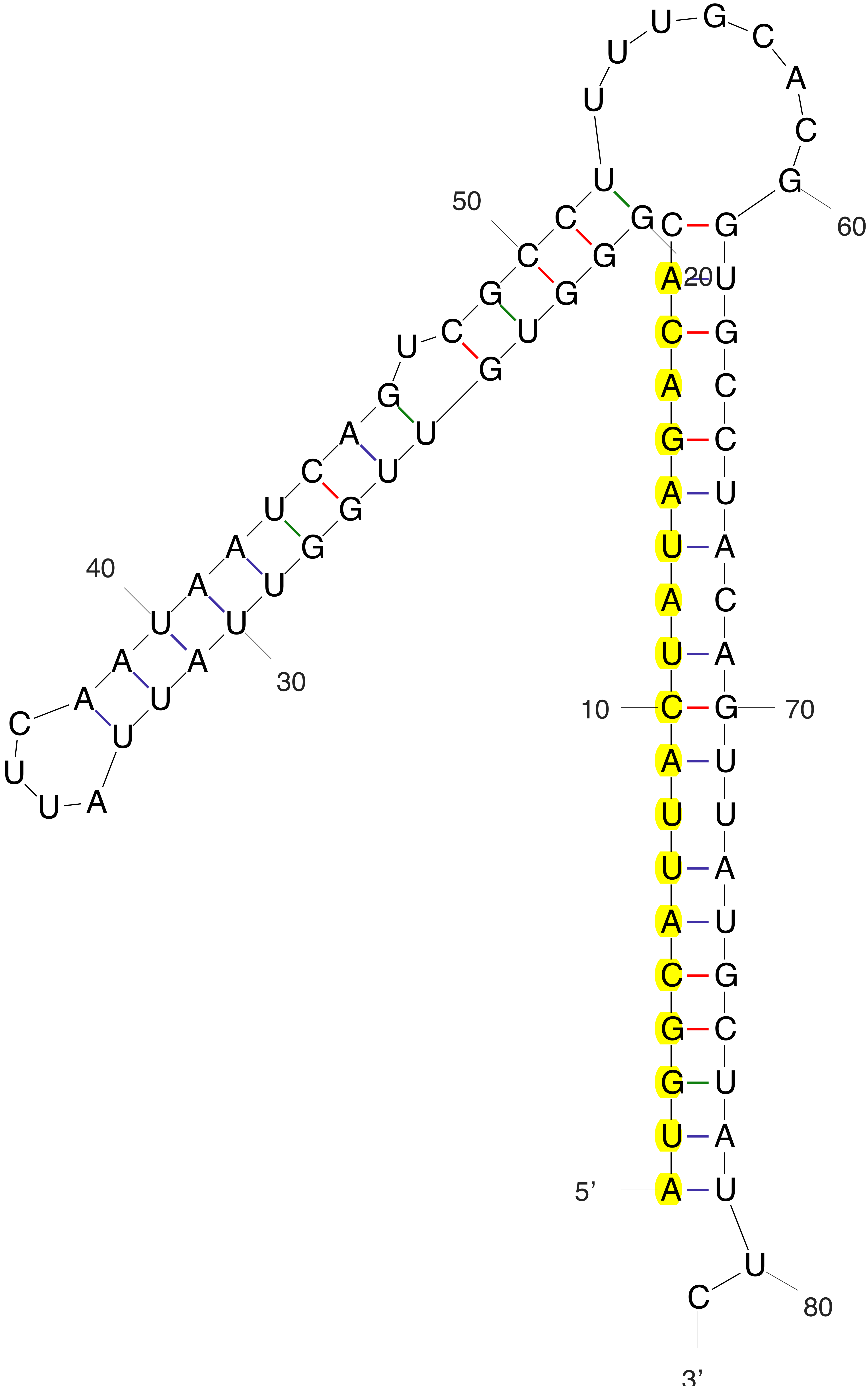

dG = -25.80 [Initially -25.80] sar-miR11

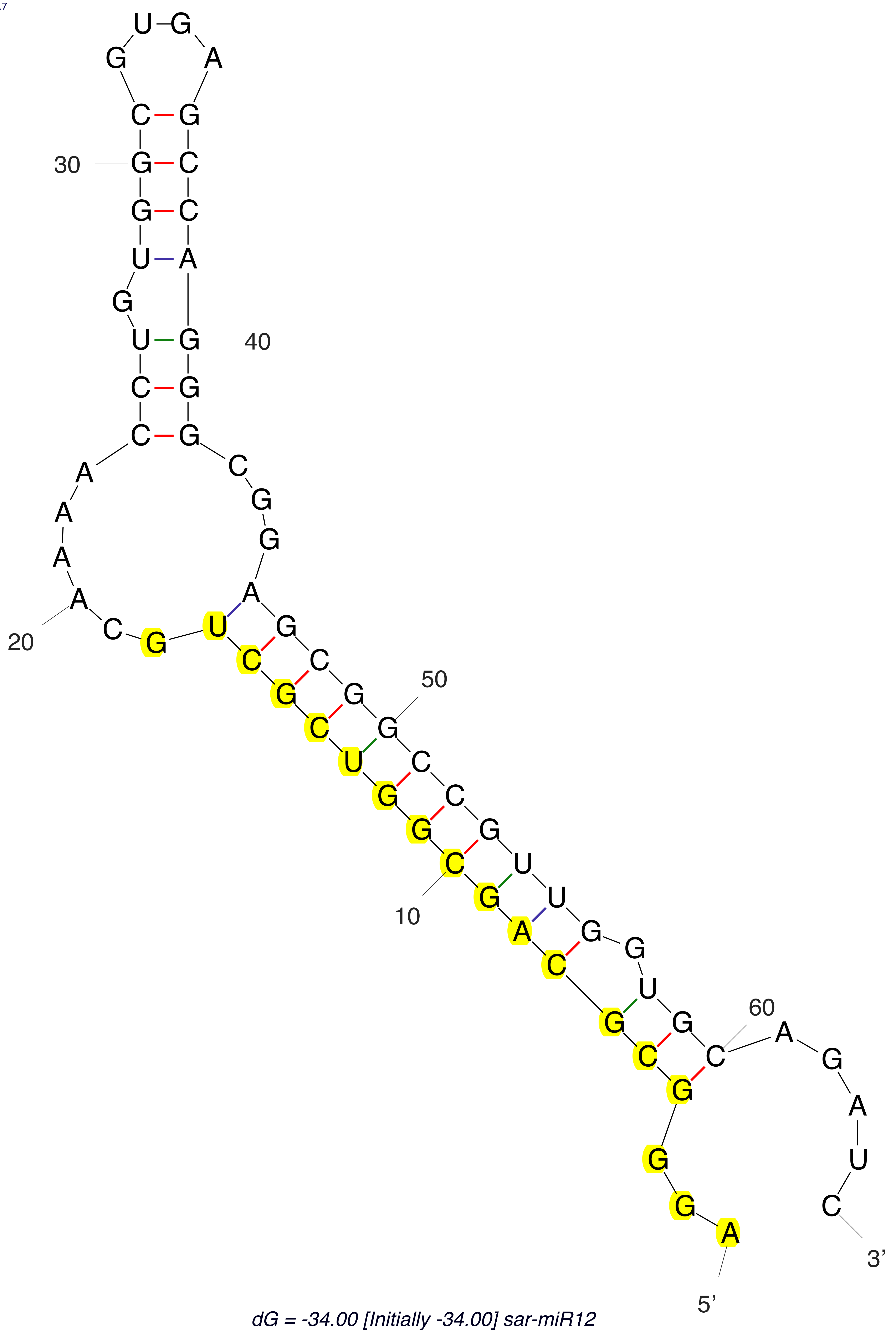

dG = -34.00 [Initially -34.00] sar-miR12

Output of sir_graph (©)
mfold_util 4.7

Created Tue Aug 23 22:28:24 2016

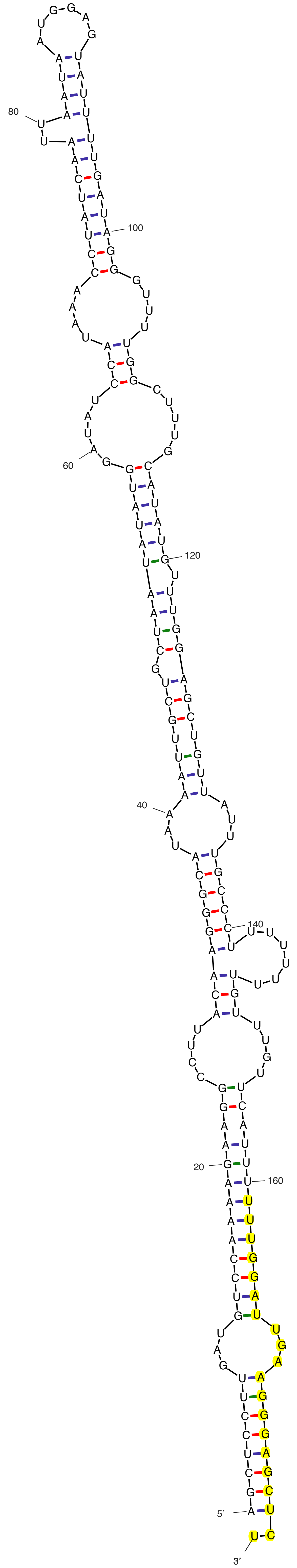

dG = -58.40 [Initially -58.40] sar-miR13

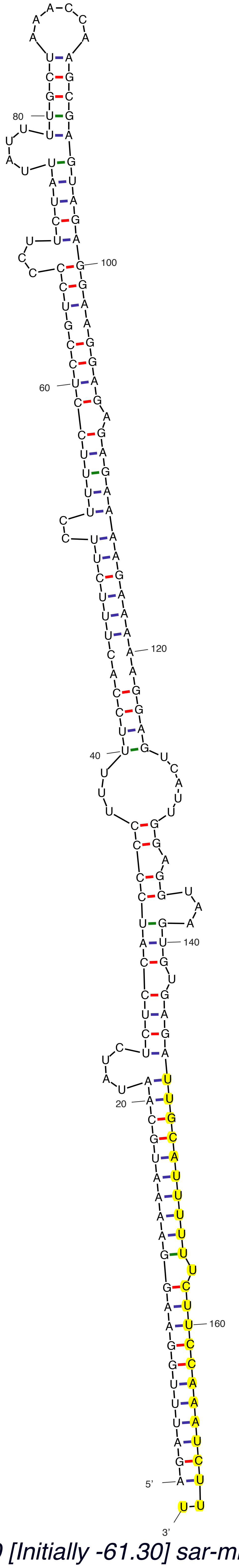

dG = -61.30 [Initially -61.30] sar-miR14

Output of sir_graph (©)
mfold_util 4.7

Created Tue Aug 23 22:41:58 2016

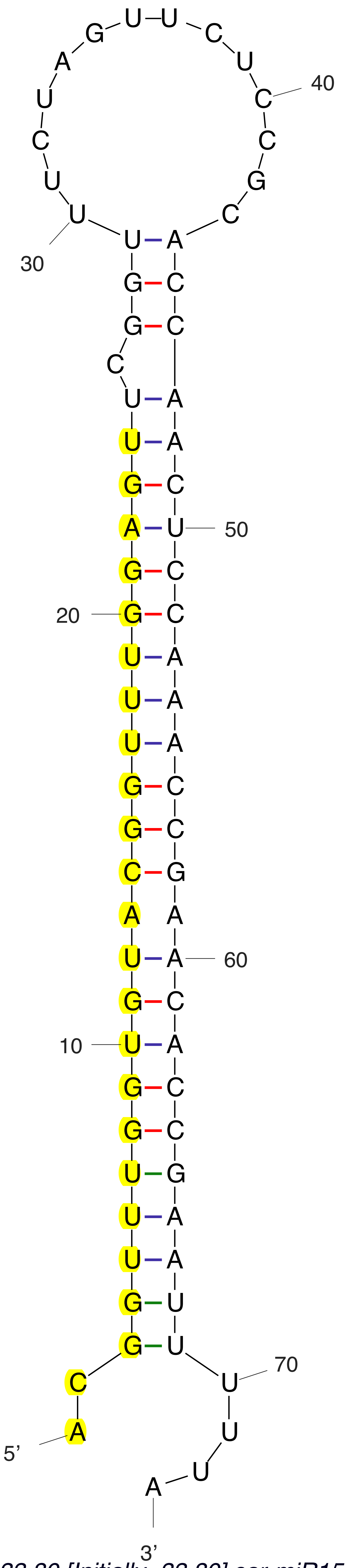

dG = -33.80 [Initially -33.80] sar-miR15

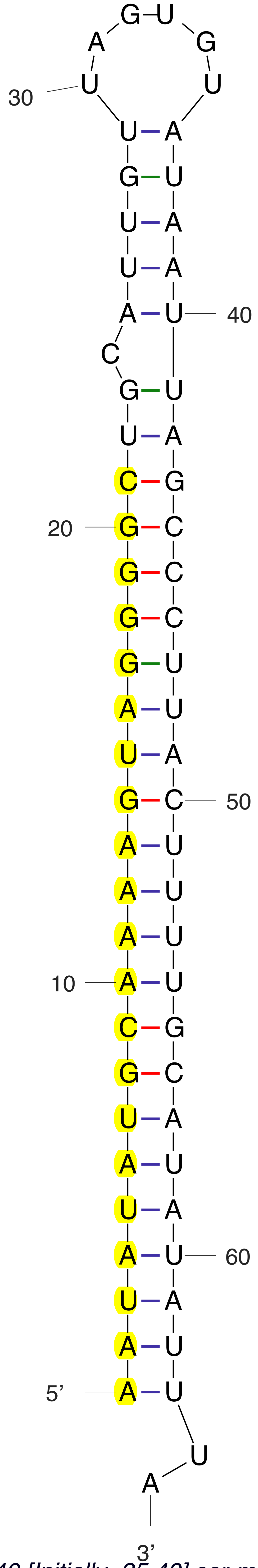

dG = -35.40 [Initially -35.40] sar-miR16

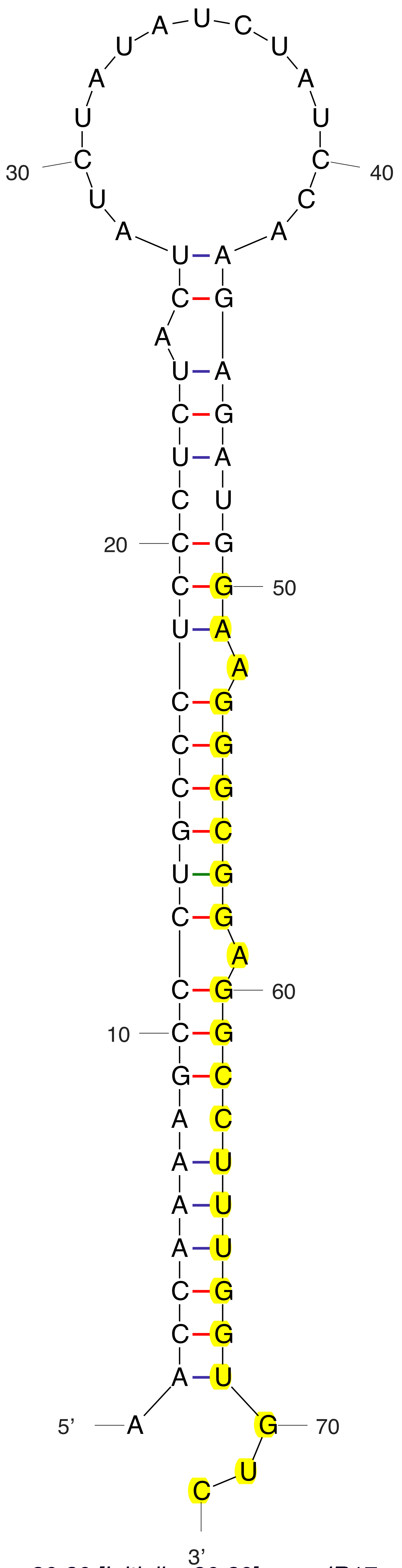

dG = -30.20 [Initially -30.20] sar-miR17

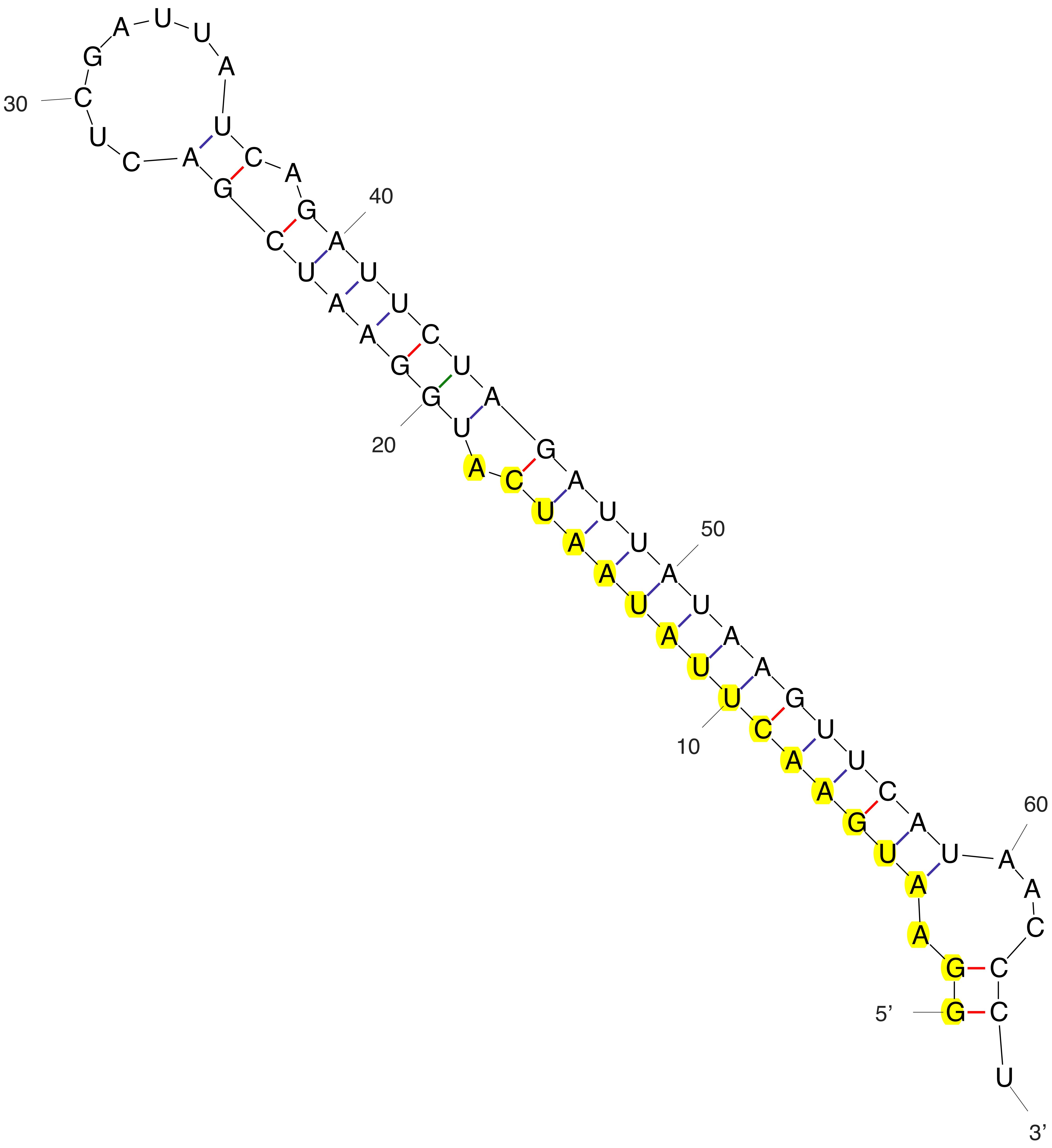

dG = -24.60 [Initially -24.60] sar-miR18

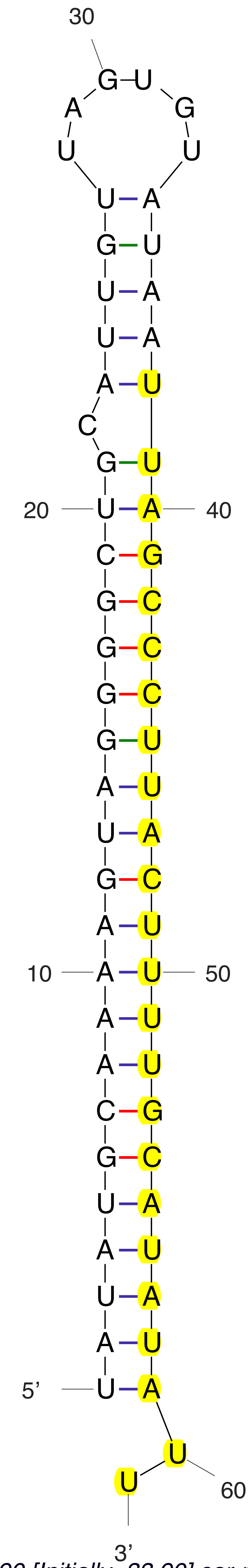

dG = -33.90 [Initially -33.90] sar-miR19

Output of sir_graph (©)
mfold_util 4.7

Created Tue Aug 23 23:37:45 2016

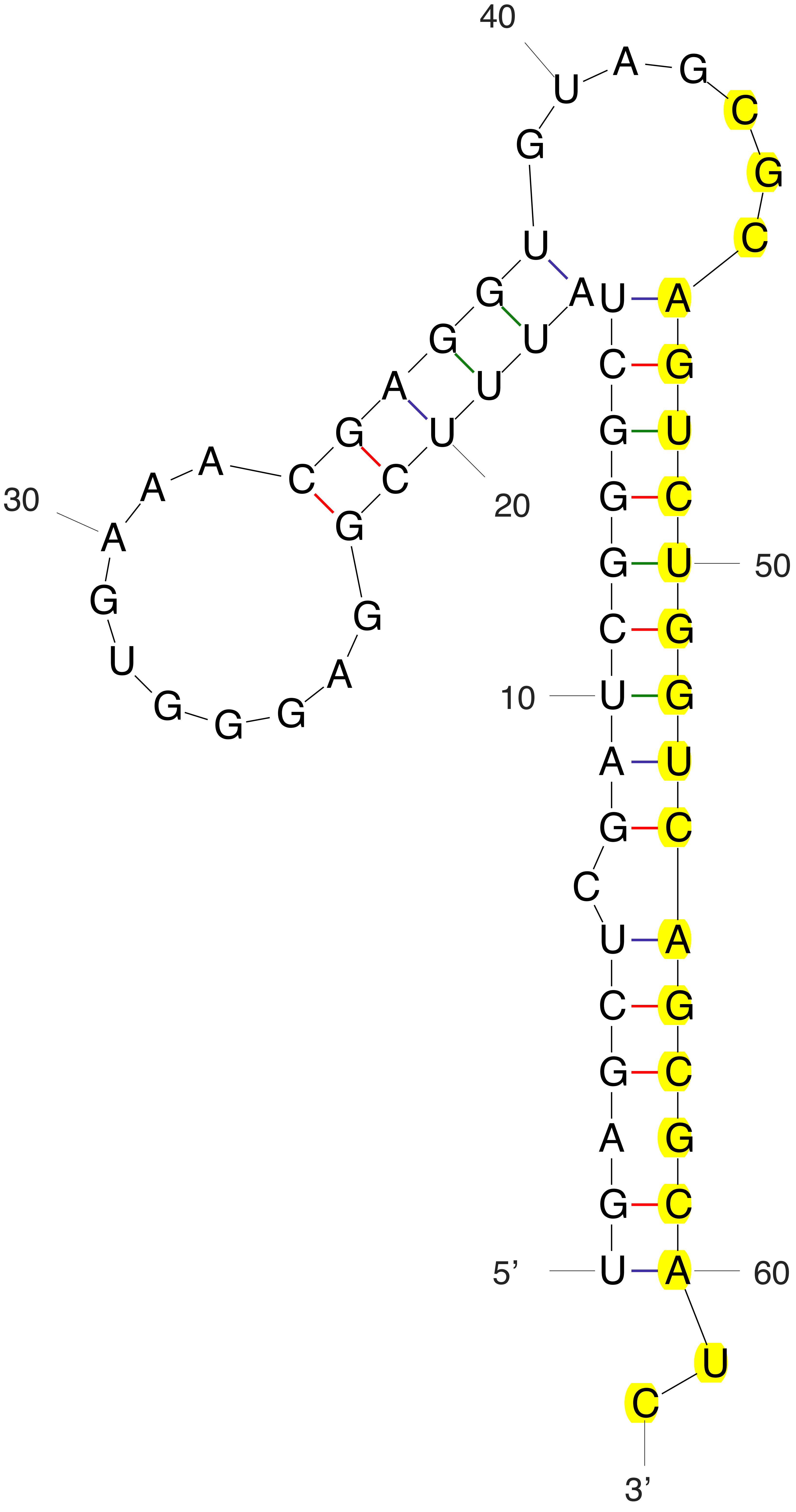

dG = -18.60 [Initially -18.60] sar-miR20
